# Supplementary material for: Boron Trifluoride Etherate Promoted Regioselective 3-Acylation of Indoles with Anhydrides
Source: Molecules. 2022 Nov 28;27(23):8281. doi: 10.3390/molecules27238281 (PMC9741063; doi:10.3390/molecules27238281)
Supplement: Supplementary file 1 [file molecules-27-08281-s001.zip › molecules-2046215-supplementary.pdf]

SUPPORTING INFORMATION

Boron Trifluoride Etherate Promoted Regioselective 3-Acylation of Indoles with Anhydrides

Yunyun Zheng, Jiuling Li * and Kai Wei *

Henan Engineering Research Center of Funiu Mountain's Medical Resources Utilization and Molecular Medicine, School of Medical Sciences, Pingdingshan University, Pingdingshan 467000, China

* Correspondence: orgchem90@163.com (J.L.); weikai1987@126.com (K.W.)

Optimization of Lewis acids

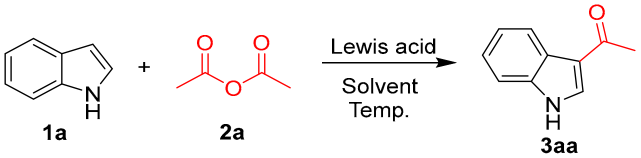

Optimization of Lewis acids

Entry	**2a** (eq.)	Lewis acid (1 eq.)	Solvent	Temp.	Time (h)	Yield (%)
1	1.2	$Sm(OTf)_3$	DCM	rt	2	nr
2	1.2	$Ce(OTf)_4$	DCM	rt	2	nr
3	1.2	$FeCl_3$	DCM	rt	2	75
4	1.2	$Fe(OTf)_3$	DCM	rt	2	60
5	1.2	$SmCl_3$	DCM	rt	2	np
6	1.2	$CeCl_3$	DCM	rt	2	np
7	1.2	$InCl_3$	DCM	rt	2	65
8	1.2	$CuCl_2$	DCM	rt	2	35
9	1.2	$BF_3{\cdot}Et_2O$	DCM	rt	2	81

Reaction conditions unless noted otherwise: **1a** (0.5 mmol), solvent (1.5 mL), np: no product, nr: no reaction.

At the beginning of this work, we have investigated different lewis acids of the reaction conditions. We could see that the acylation procedure catalyzed by $FeCl_3$, $Fe(OTf)_3$ and $InCl_3$ could give the 3-acylindole in 75%, 60% and 65% yields, respectively (Entries 3, 7 and 4). This reaction had a yield of only 35% when catalyzed by $CuCl_2$. In addition, Under the catalysis of $Sm(OTf)_3$ and $Ce(OTf)_4$, the reaction could not occur. No desired product was achieved catalyzed by $SmCl_3$ and $CeCl_3$. The yield of 3-acylindole **3aa** achieved 81% in the presence of $BF_3{\cdot}Et_2O$.

^{1}H NMR spectra (500 MHz, DMSO-d_6) of 3aa

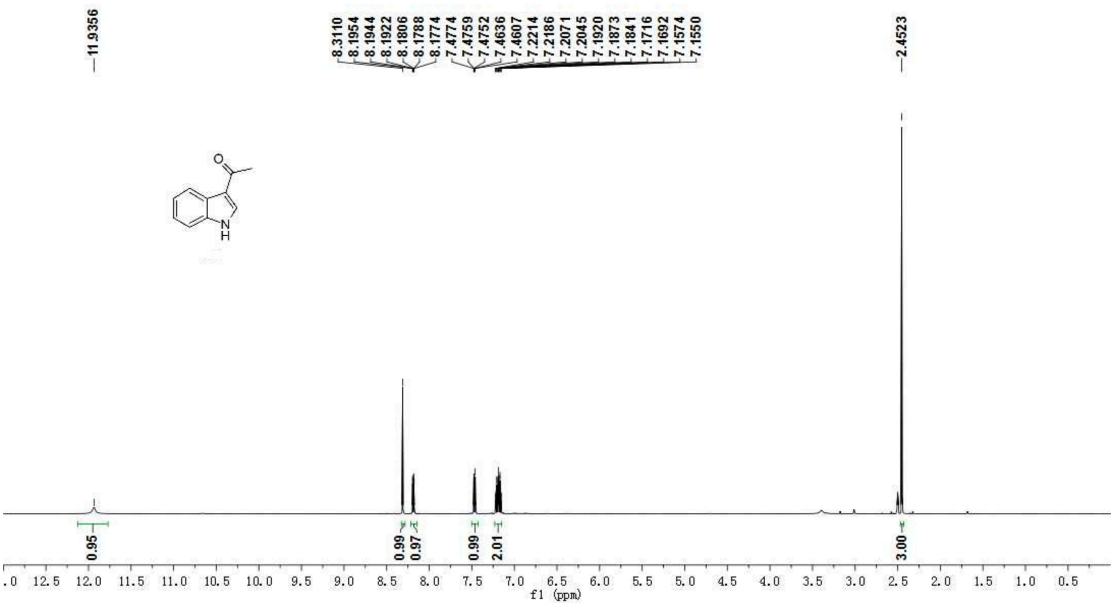

^{13}C NMR spectra (125 MHz, DMSO-d_6) of 3aa

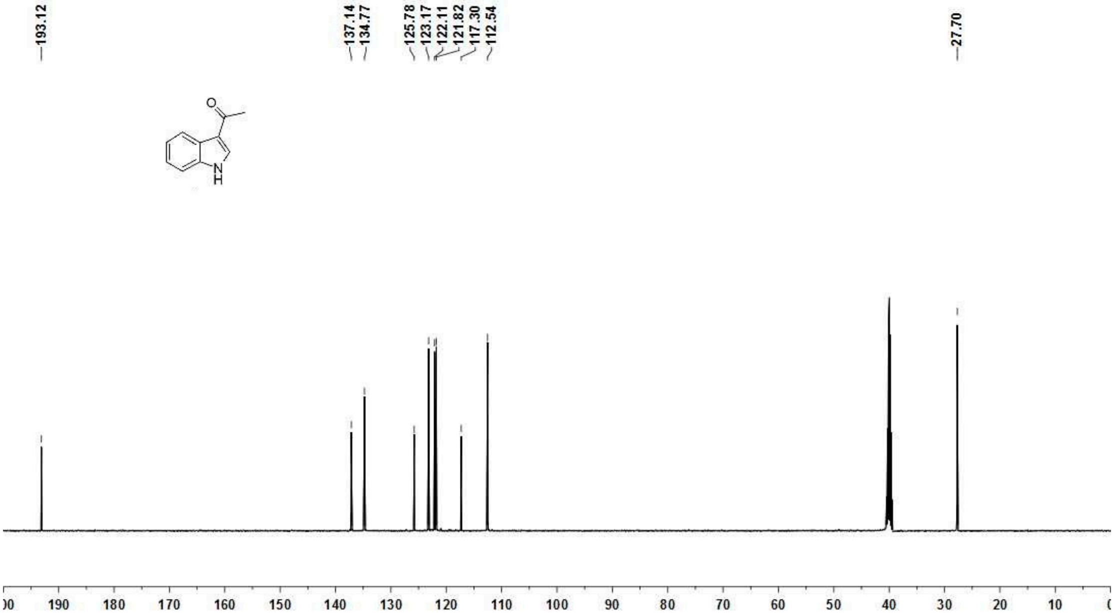

^{1}H NMR spectra (500 MHz, DMSO-d_6) of 3ab

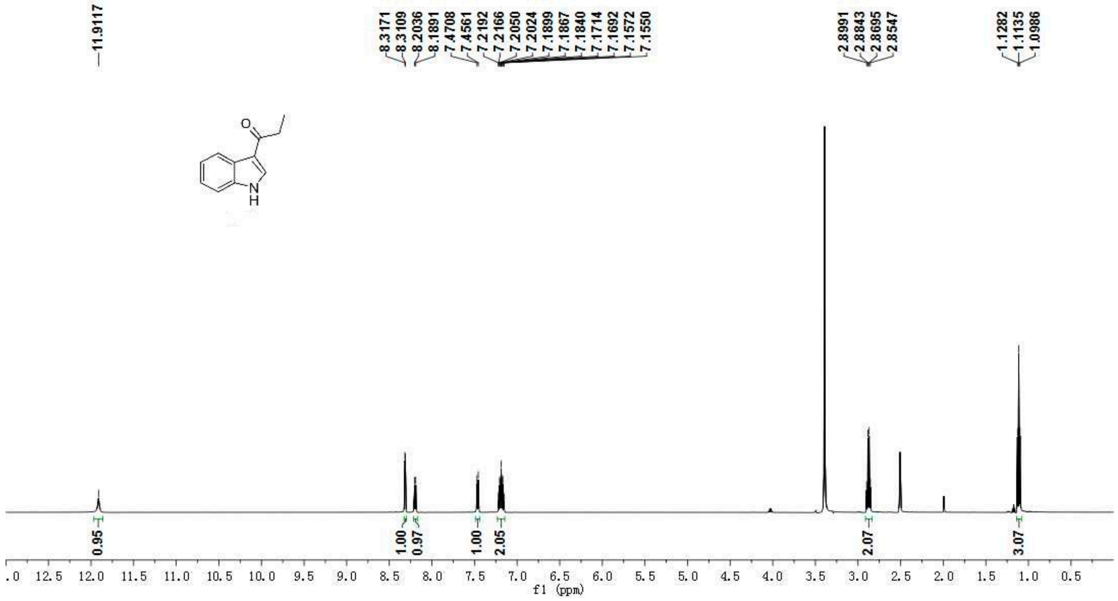

^{13}C NMR spectra (125 MHz, DMSO-d_6) of 3ab

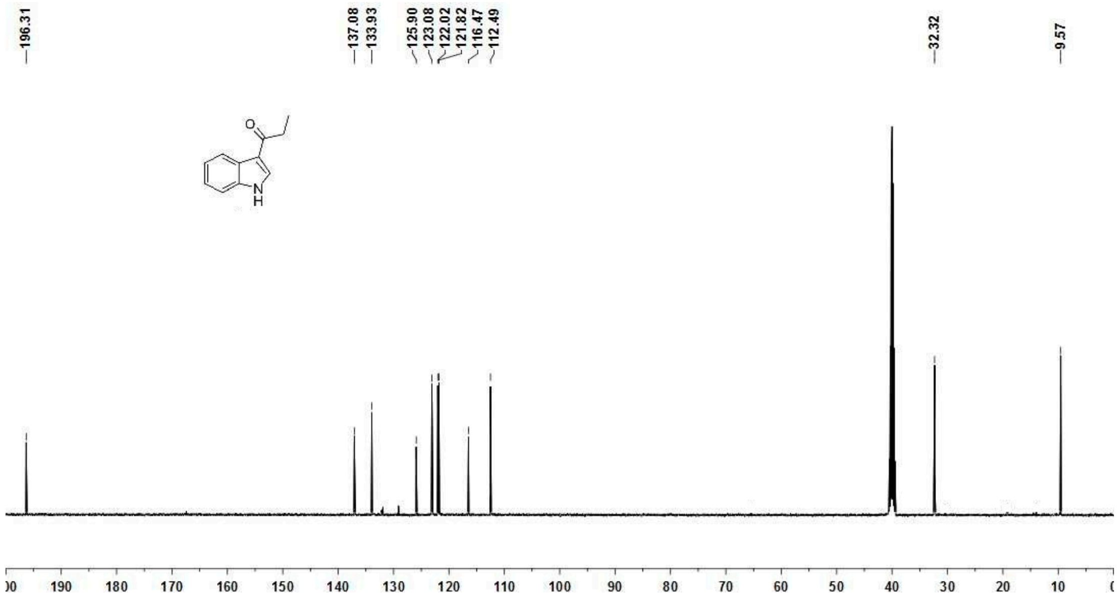

^{1}H NMR spectra (500 MHz, DMSO-d_6) of 3ac

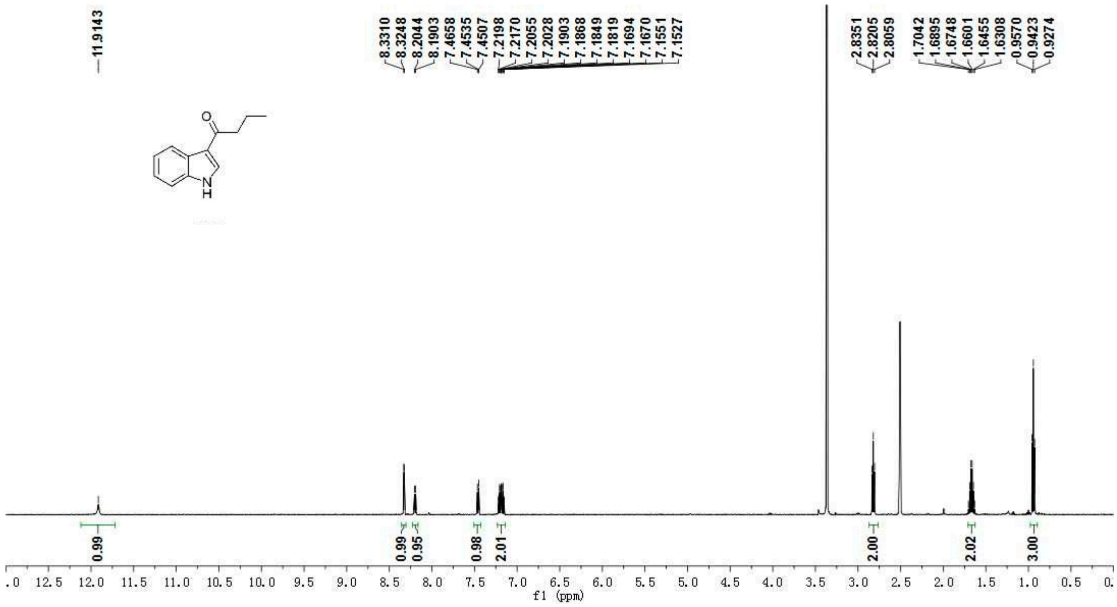

^{13}C NMR spectra (125 MHz, DMSO-d_6) of 3ac

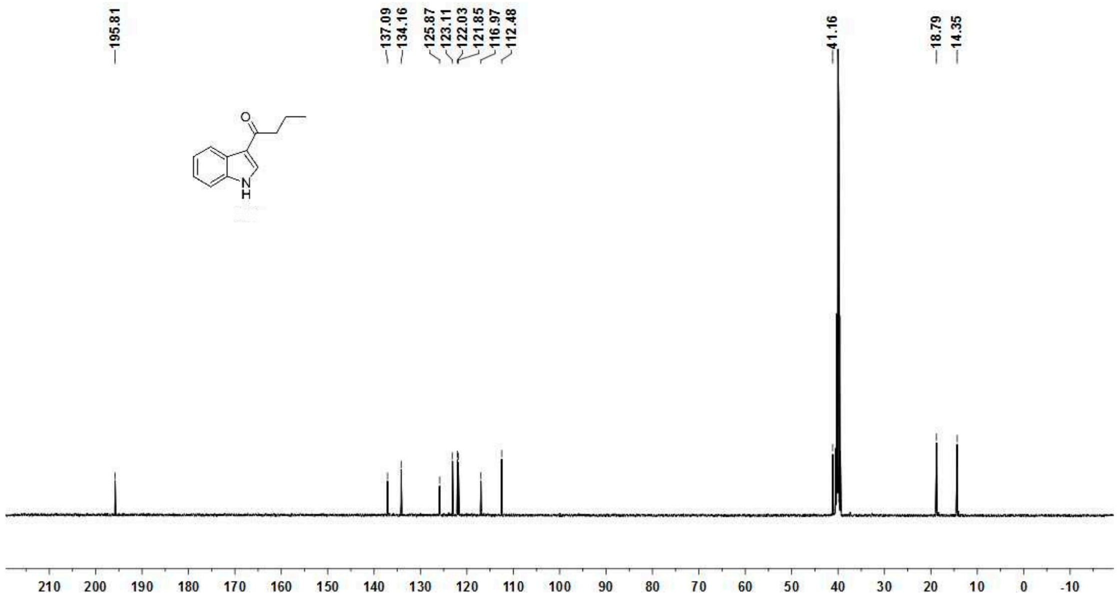

^{1}H NMR spectra (500 MHz, DMSO-d_6) of 3ad

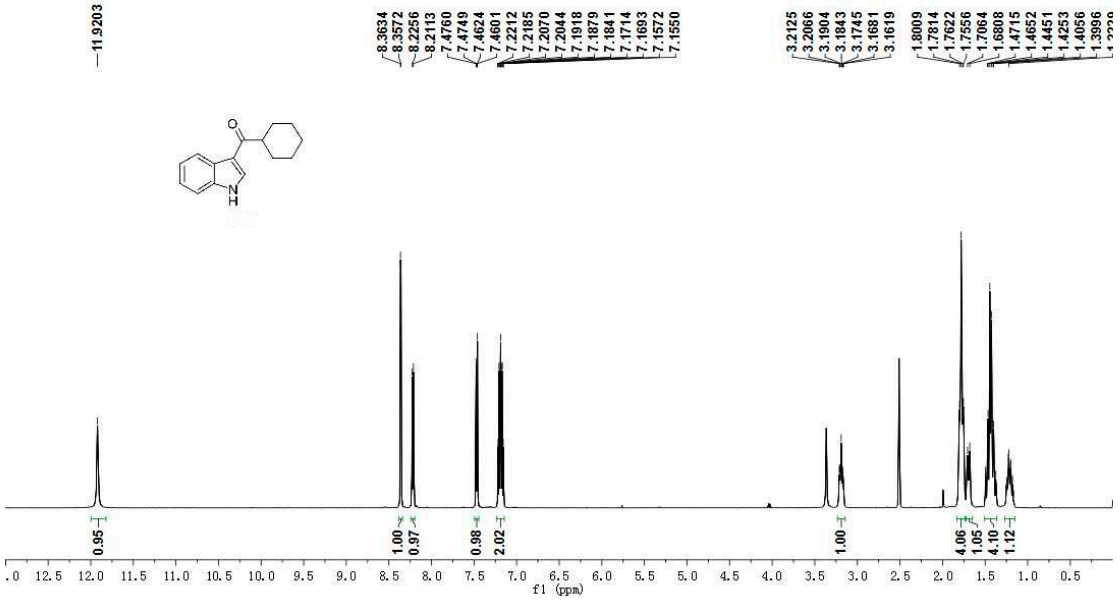

^{13}C NMR spectra (125 MHz, DMSO-d_6) of 3ad

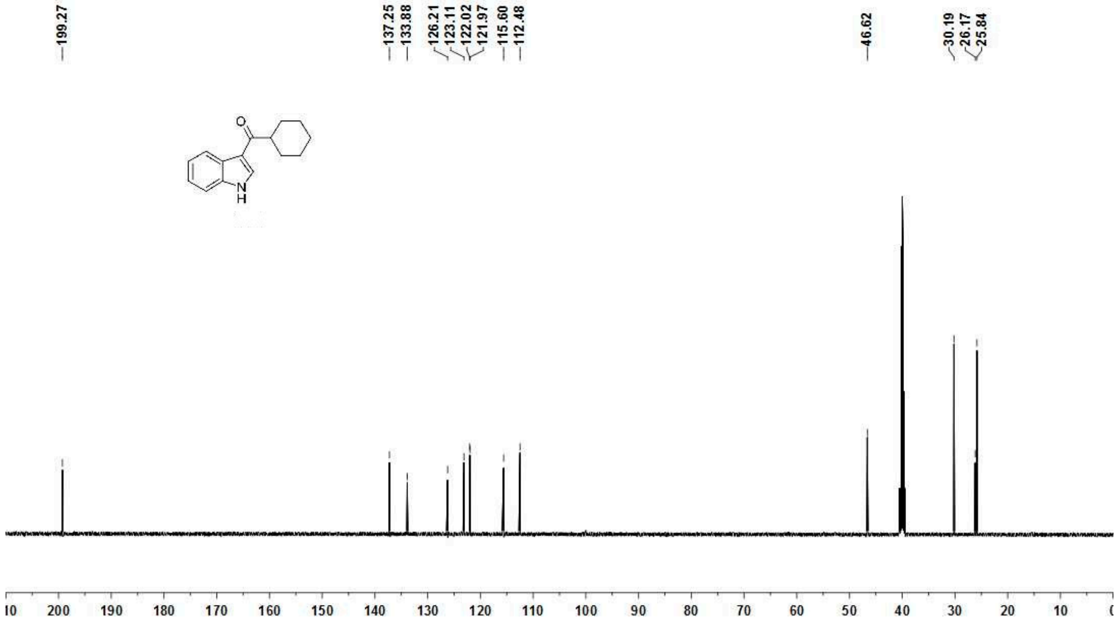

^{1}H NMR spectra (500 MHz, DMSO-d_6) of 3ae

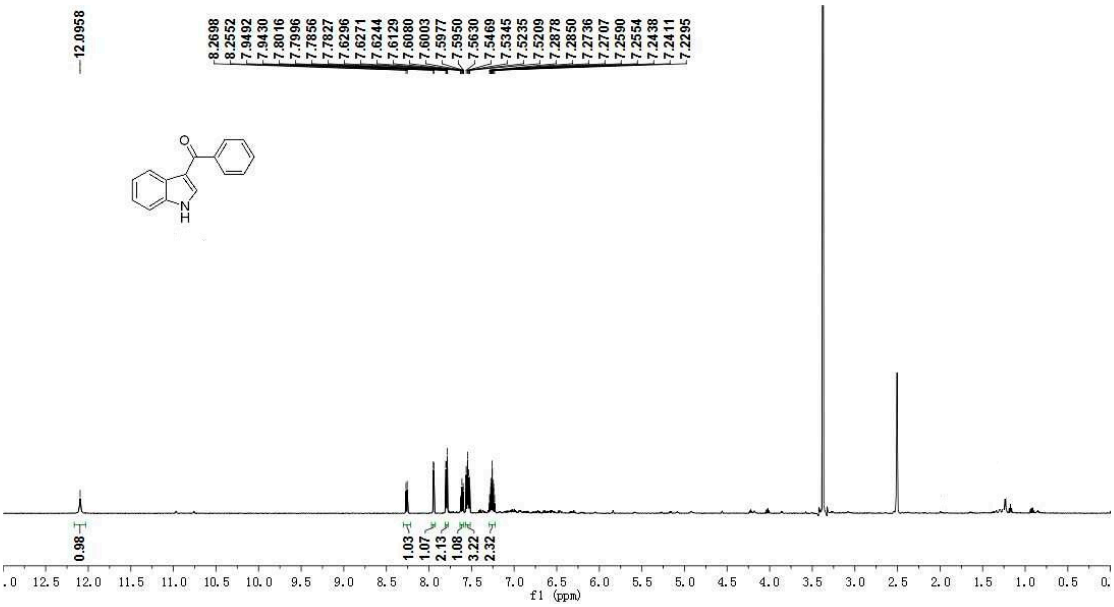

^{13}C NMR spectra (125 MHz, DMSO-d_6) of 3ae

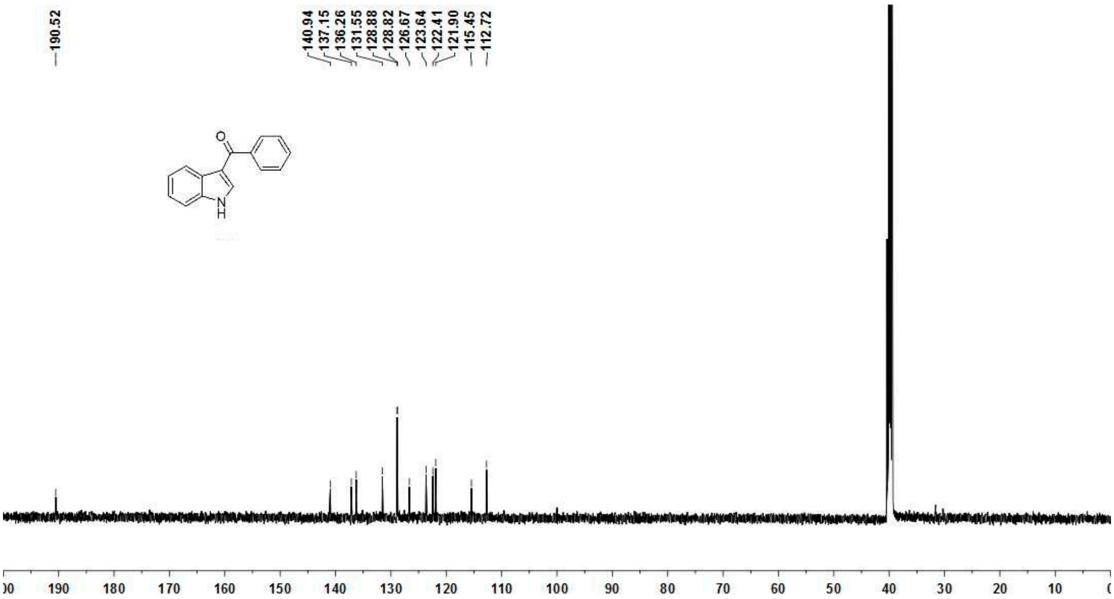

^{1}H NMR spectra (500 MHz, DMSO-d_6) of 3af

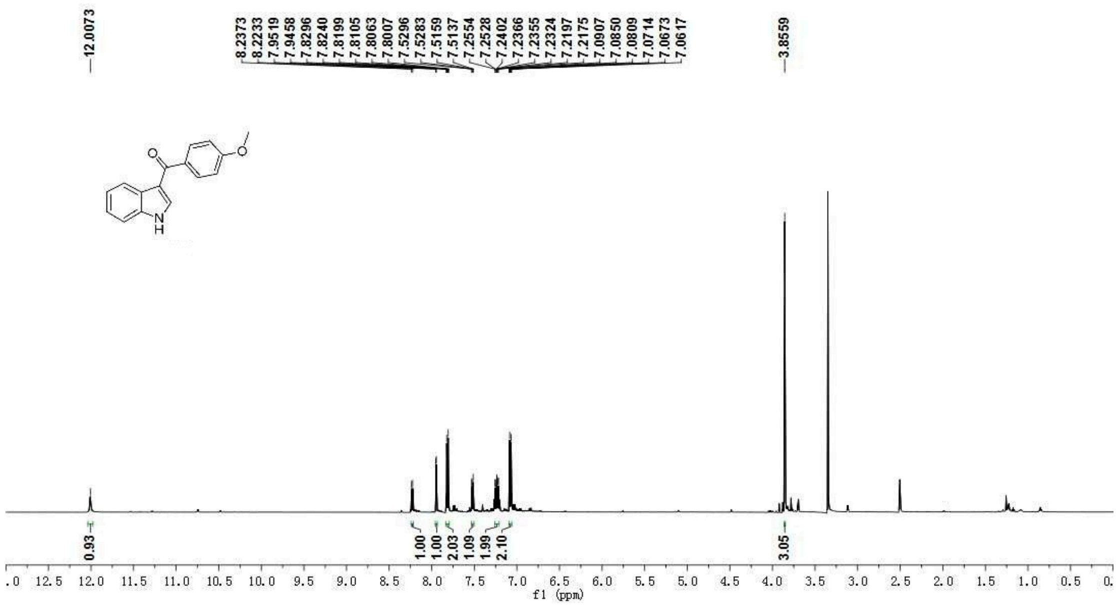

^{13}C NMR spectra (125 MHz, DMSO-d_6) of 3af

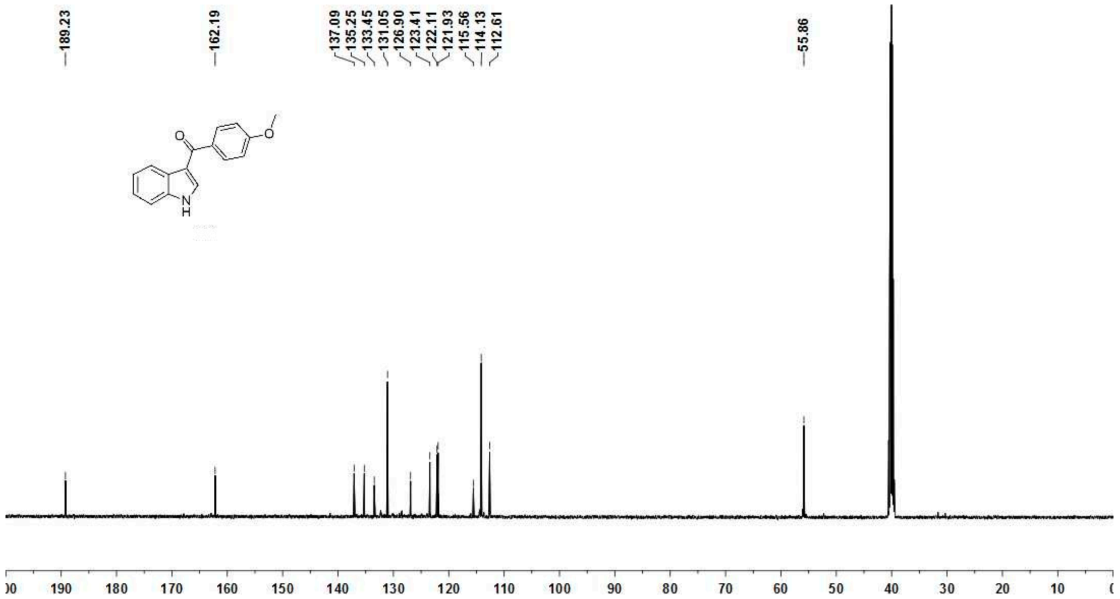

^{1}H NMR spectra (500 MHz, DMSO-d_6) of 3ag

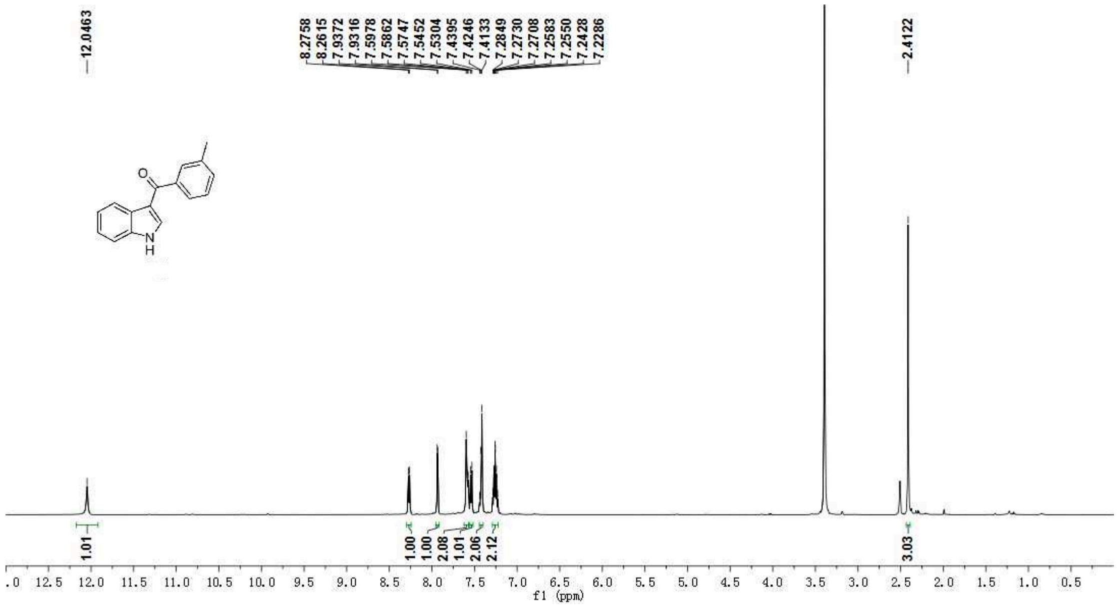

^{13}C NMR spectra (125 MHz, DMSO-d_6) of 3ag

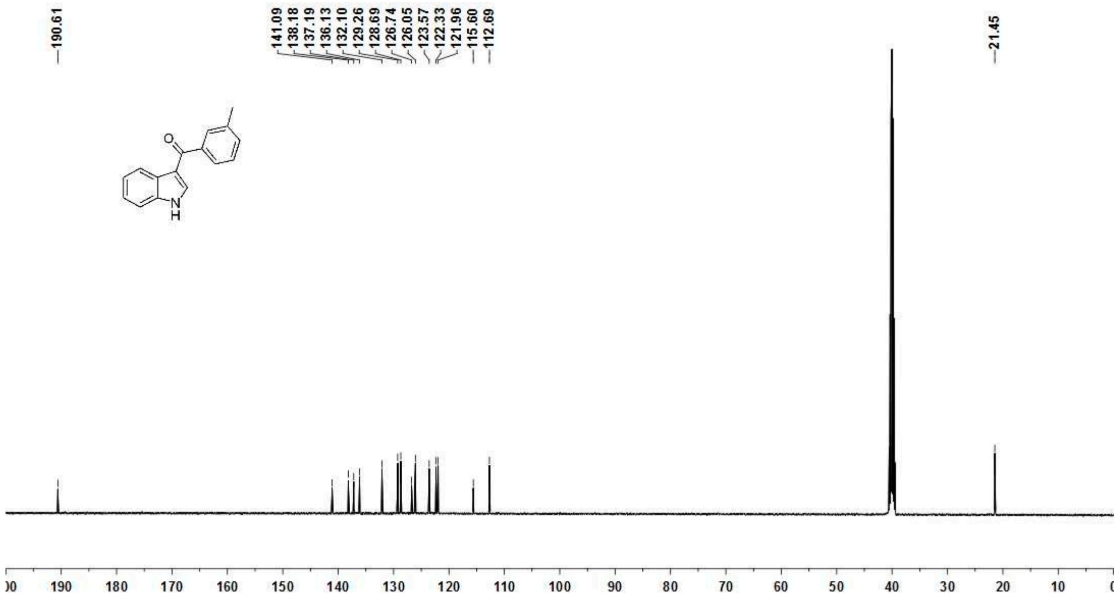

^{1}H NMR spectra (500 MHz, DMSO-d_6) of 3ba

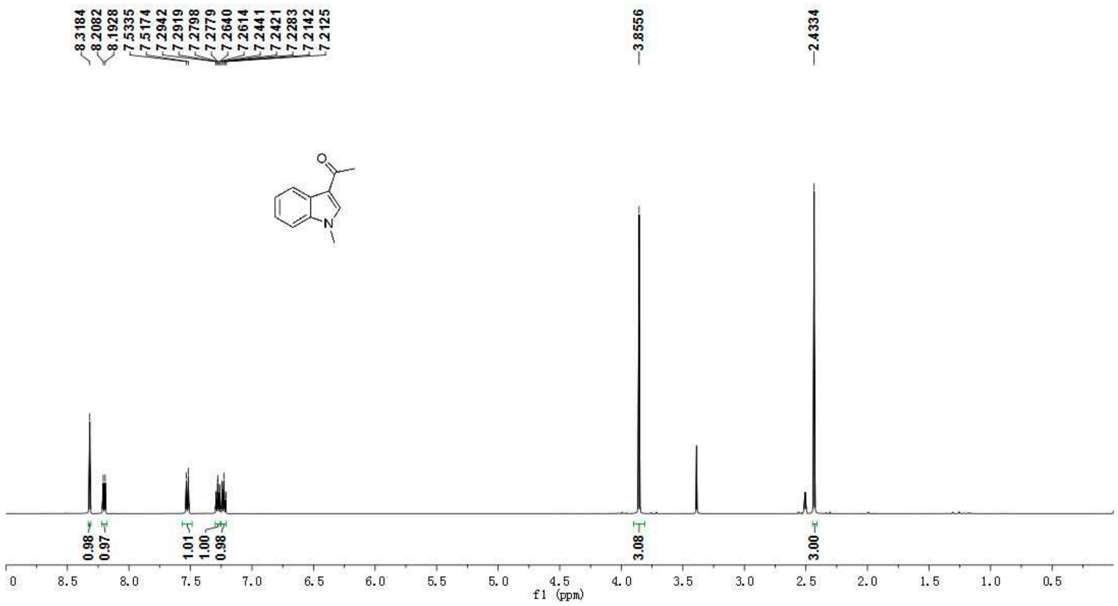

^{13}C NMR spectra (125 MHz, DMSO-d_6) of 3ba

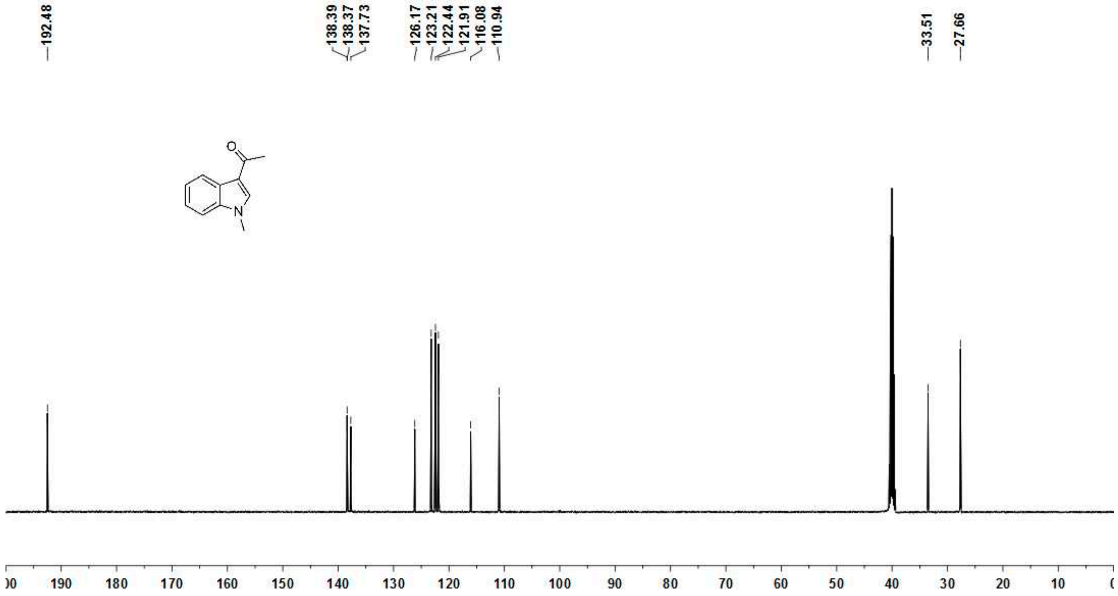

^{1}H NMR spectra (500 MHz, DMSO-d_6) of 3bb

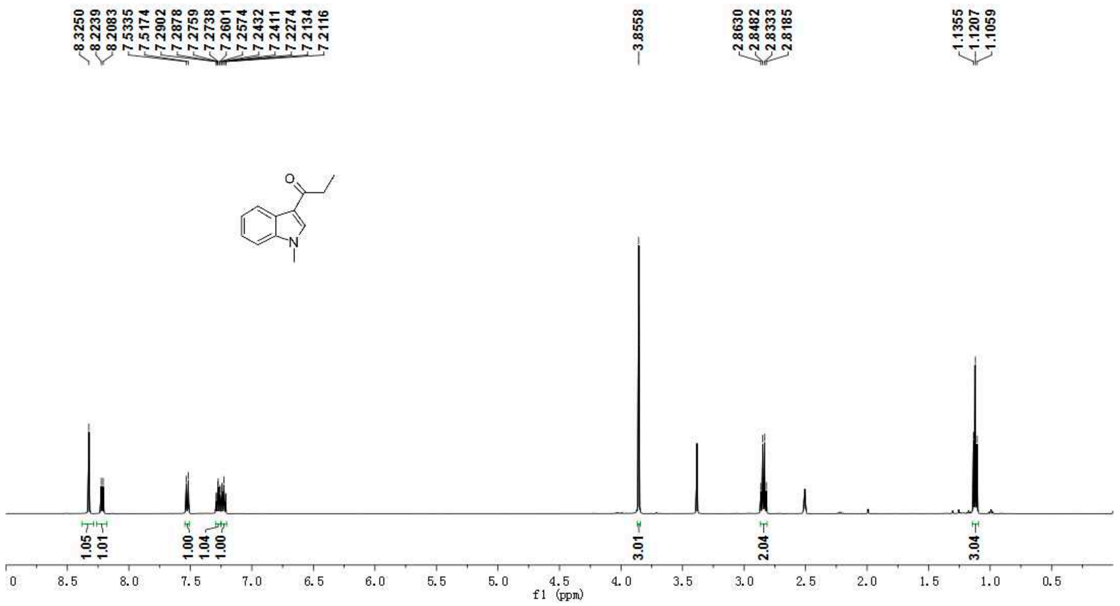

^{13}C NMR spectra (125 MHz, DMSO-d_6) of 3bb

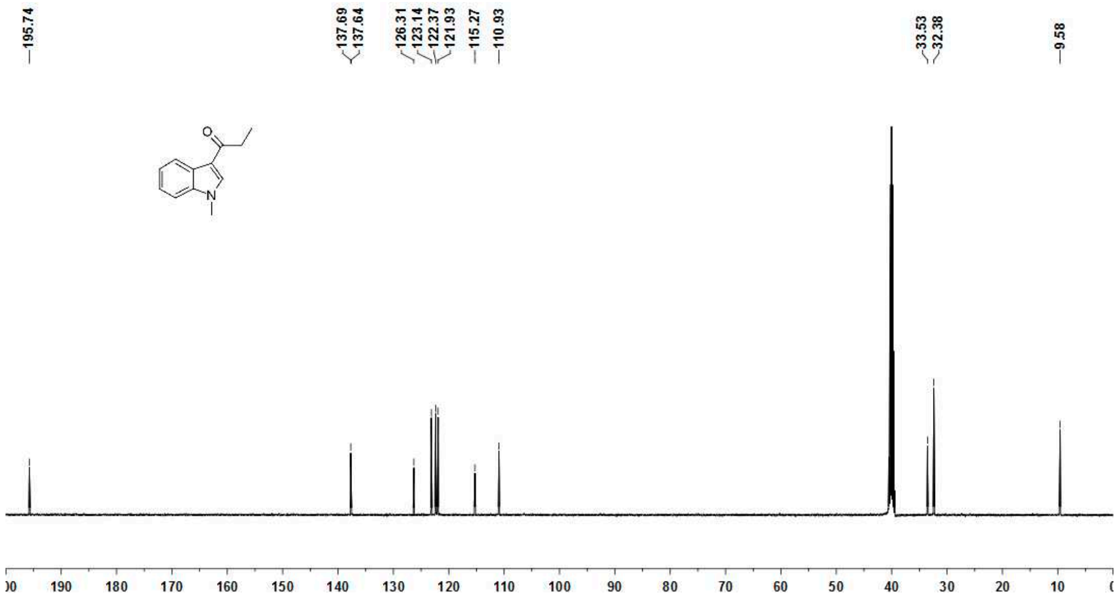

^{1}H NMR spectra (500 MHz, DMSO-d_6) of 3bc

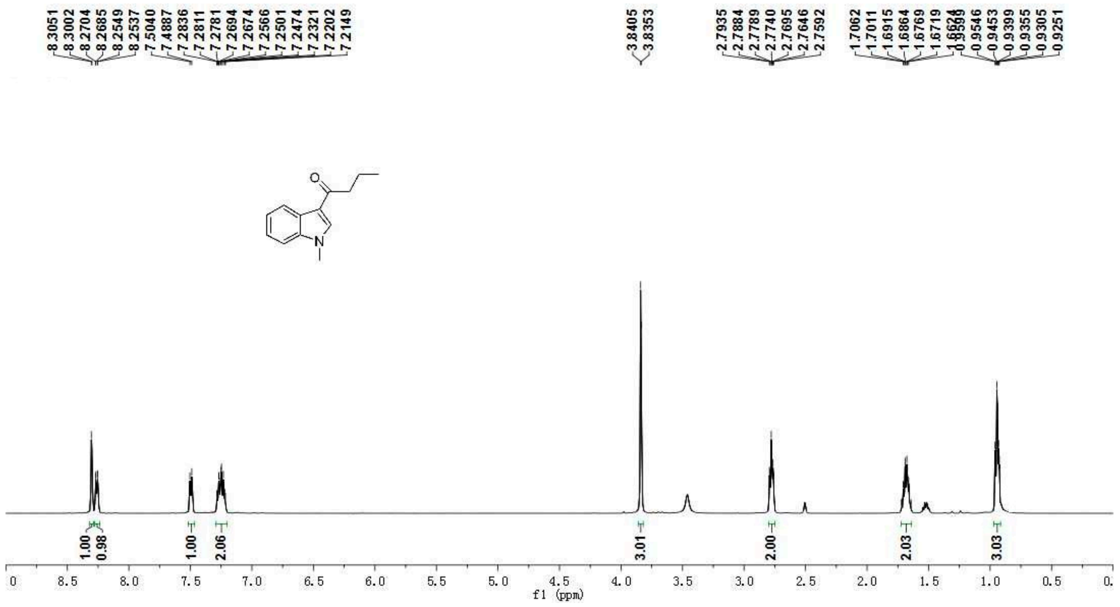

^{13}C NMR spectra (125 MHz, DMSO-d_6) of 3bc

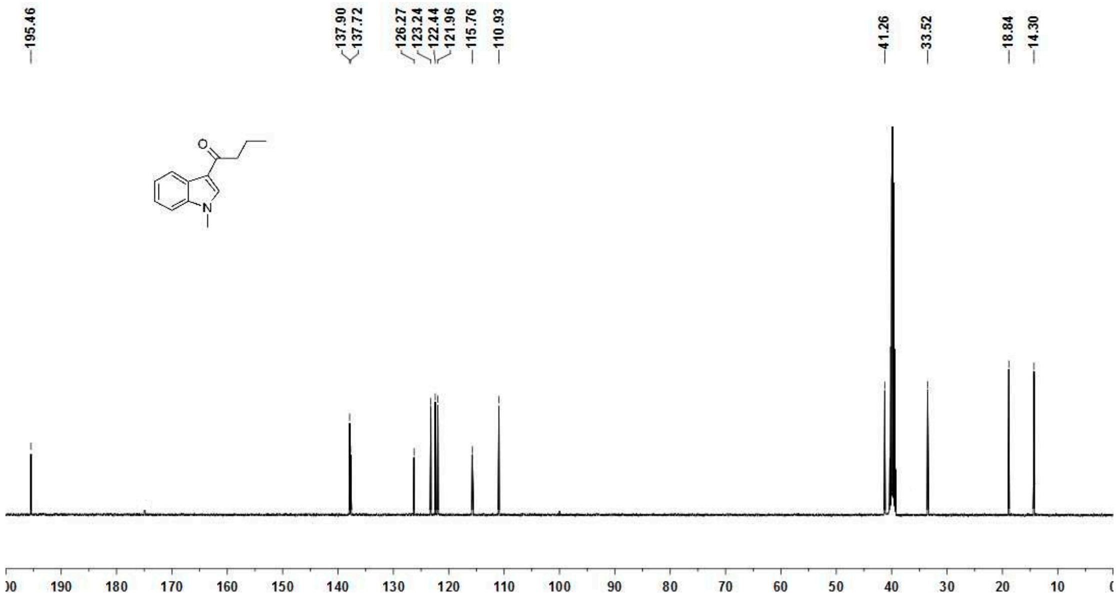

^{1}H NMR spectra (500 MHz, DMSO-d_6) of 3bd

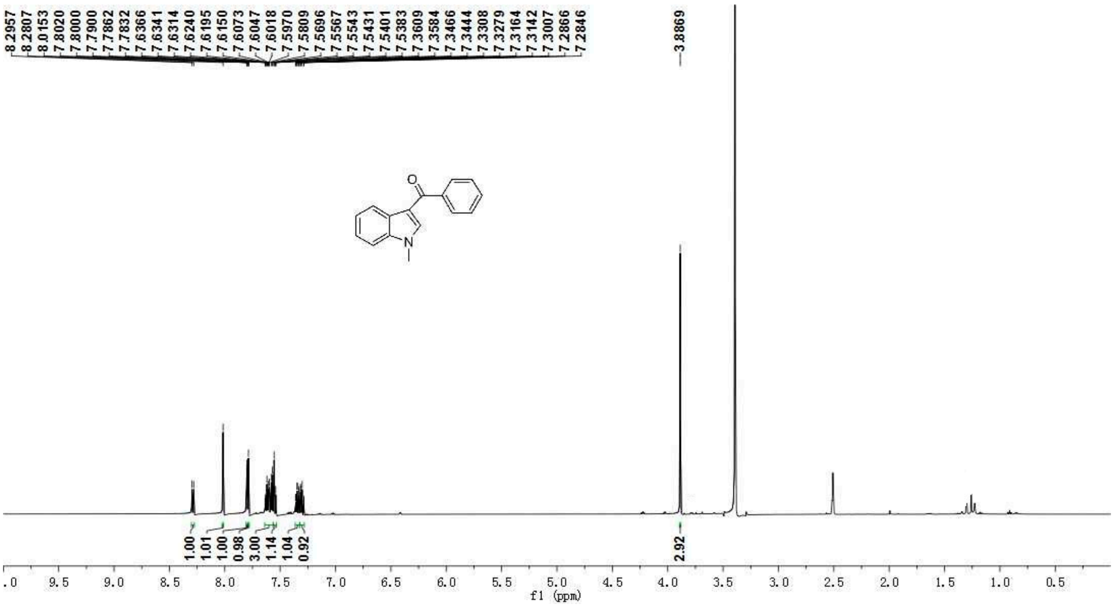

^{13}C NMR spectra (125 MHz, DMSO-d_6) of 3bd

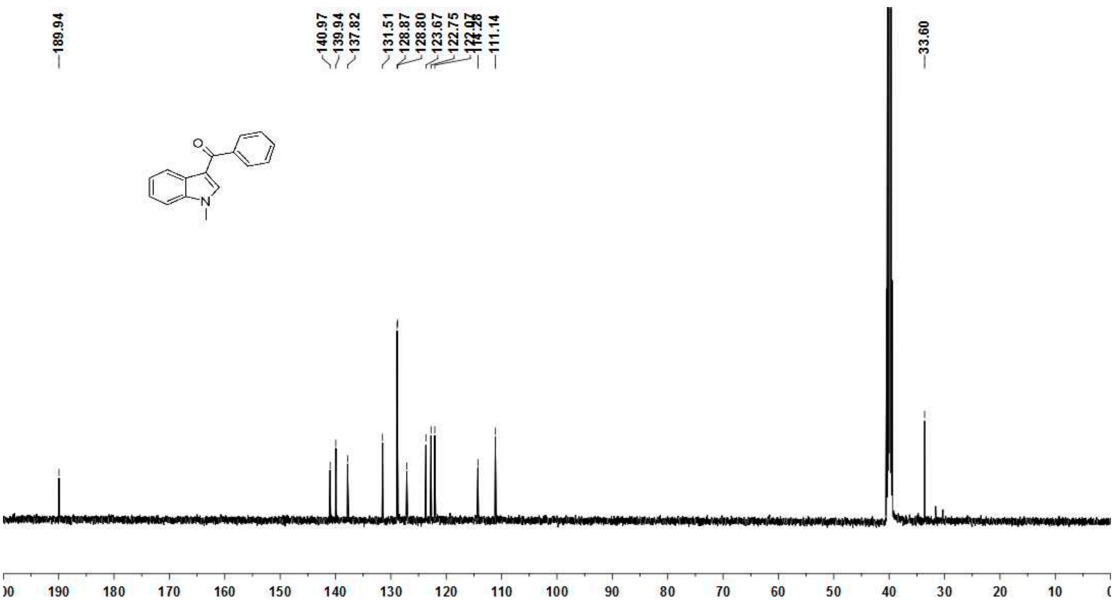

^{1}H NMR spectra (500 MHz, DMSO-d_6) of 3ca

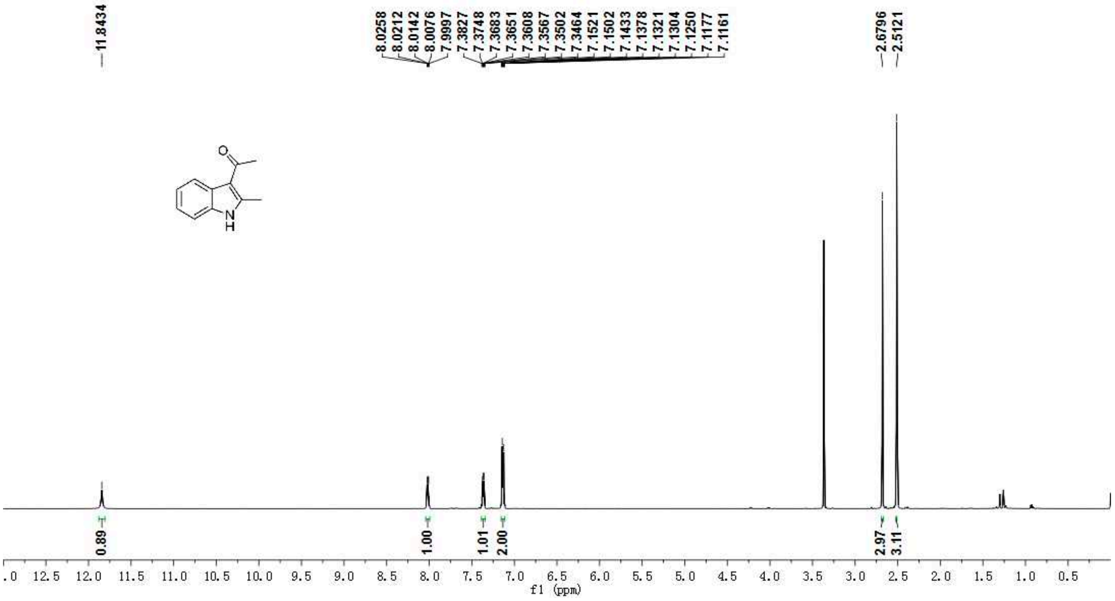

^{13}C NMR spectra (125 MHz, DMSO-d_6) of 3ca

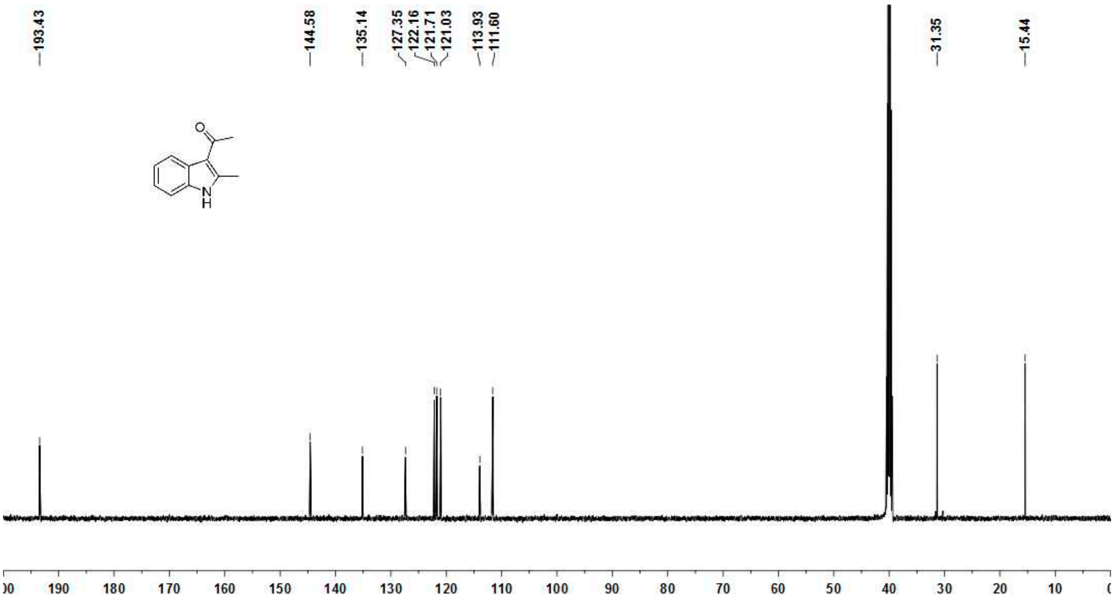

^{1}H NMR spectra (500 MHz, DMSO-d_6) of 3cb

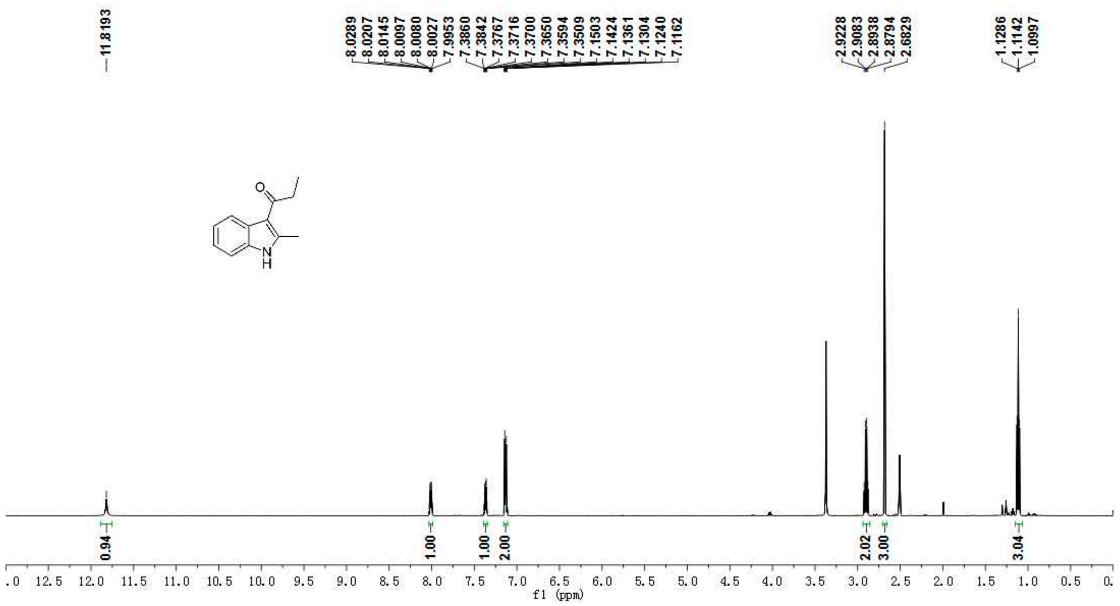

^{13}C NMR spectra (125 MHz, DMSO-d_6) of 3cb

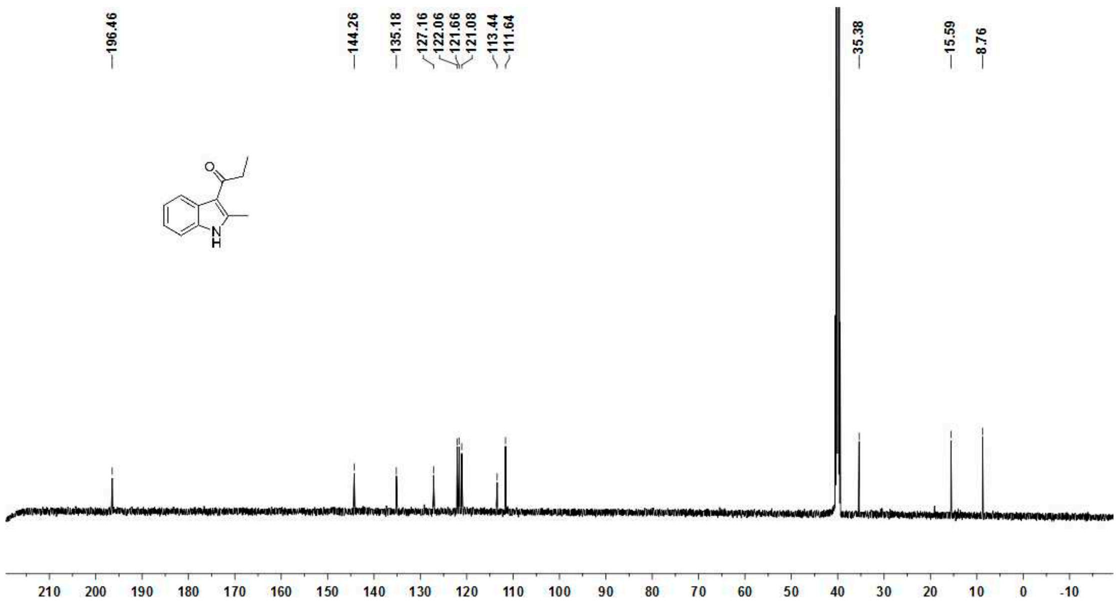

^{1}H NMR spectra (500 MHz, DMSO-d_6) of 3cc

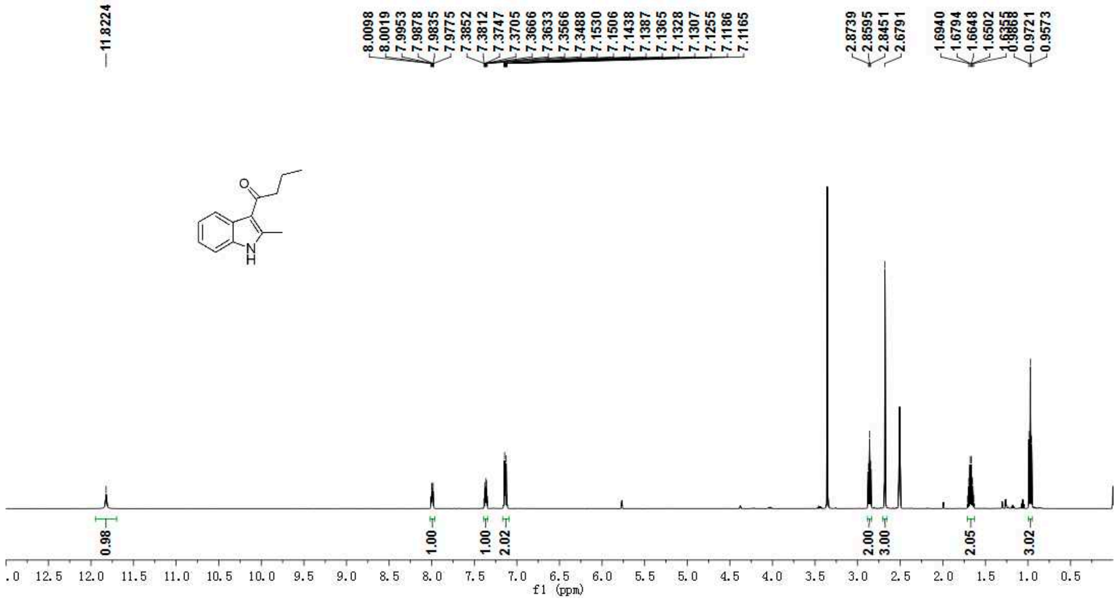

^{13}C NMR spectra (125 MHz, DMSO-d_6) of 3cc

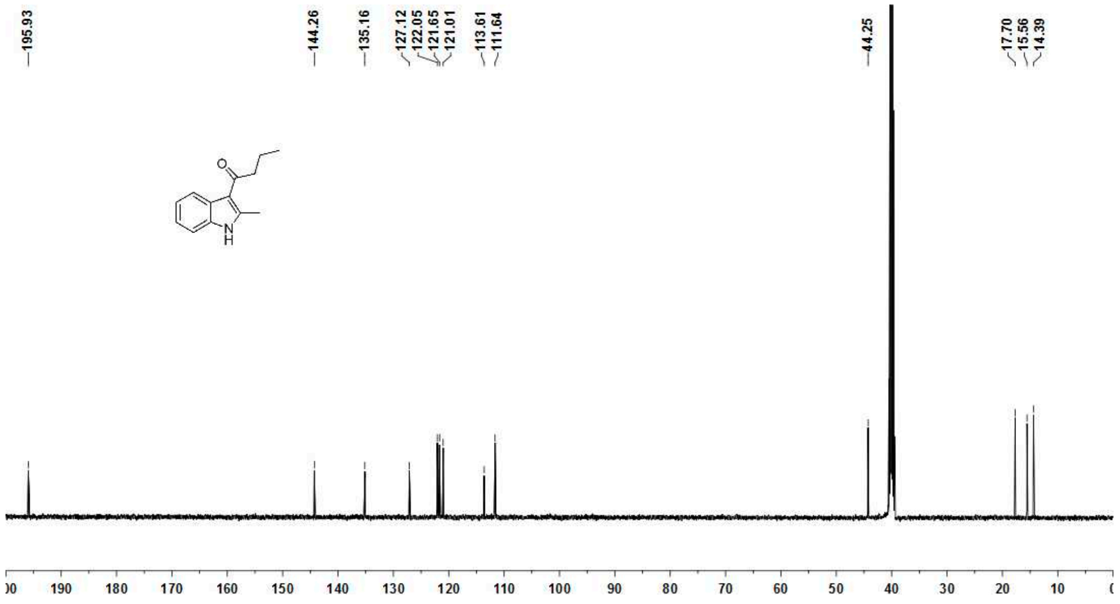

^{1}H NMR spectra (500 MHz, DMSO-d_6) of 3cd

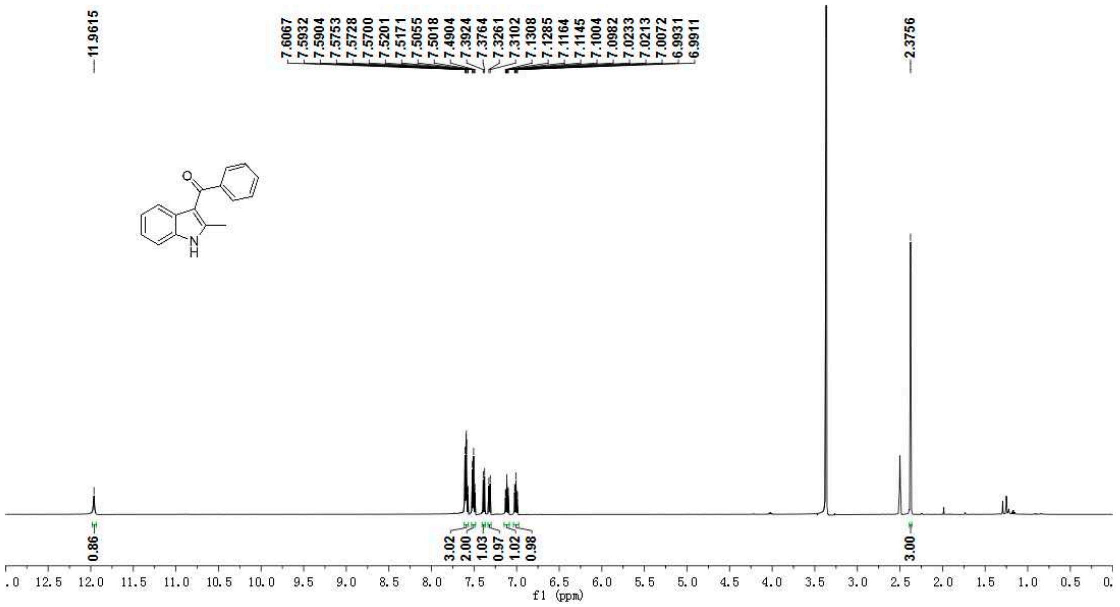

^{13}C NMR spectra (125 MHz, DMSO-d_6) of 3cd

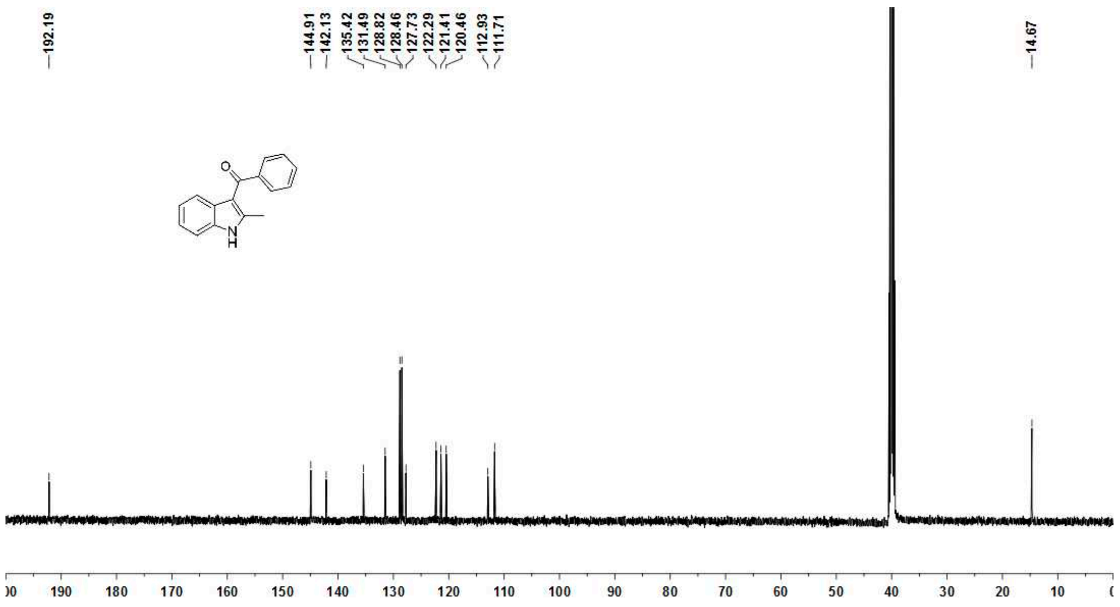

^{1}H NMR spectra (500 MHz, DMSO-d_6) of 3da

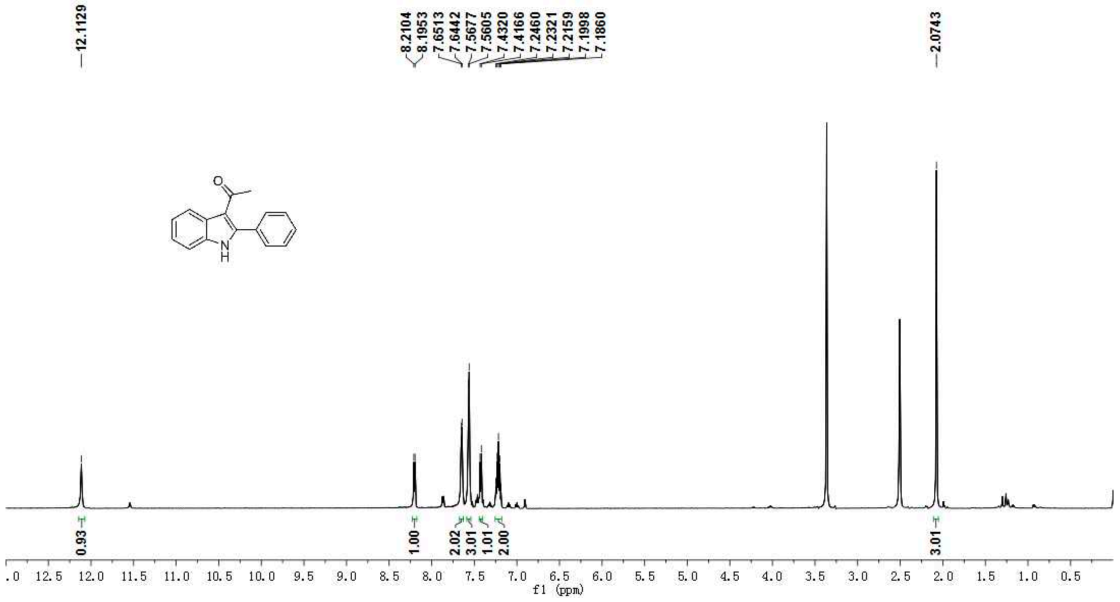

^{13}C NMR spectra (125 MHz, DMSO-d_6) of 3da

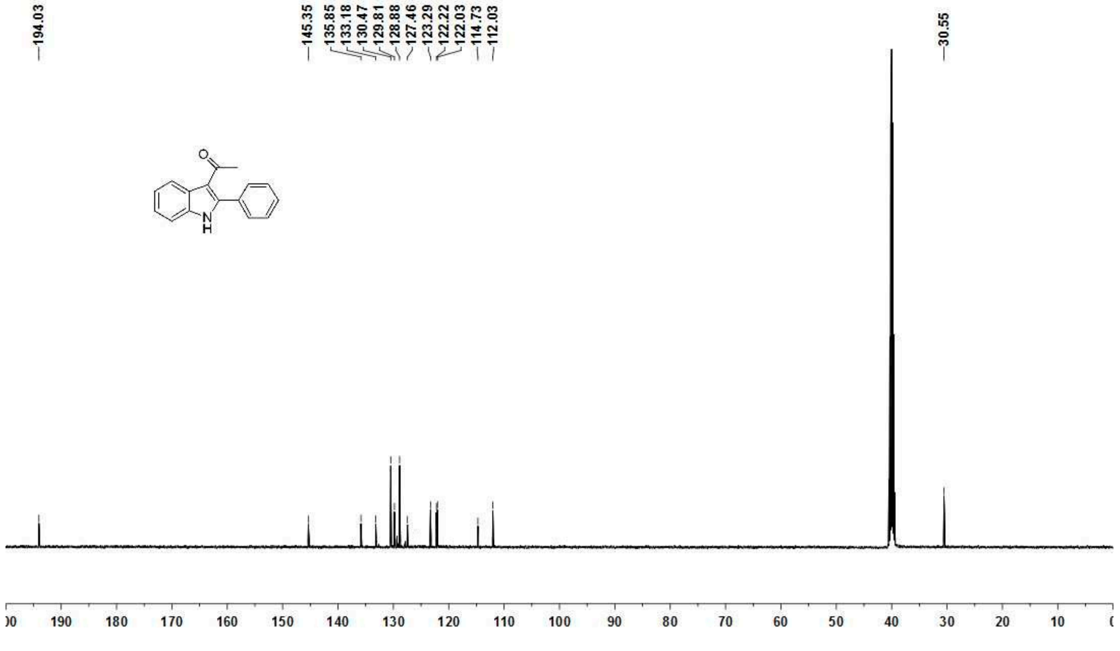

^{1}H NMR spectra (500 MHz, DMSO-d_6) of 3db

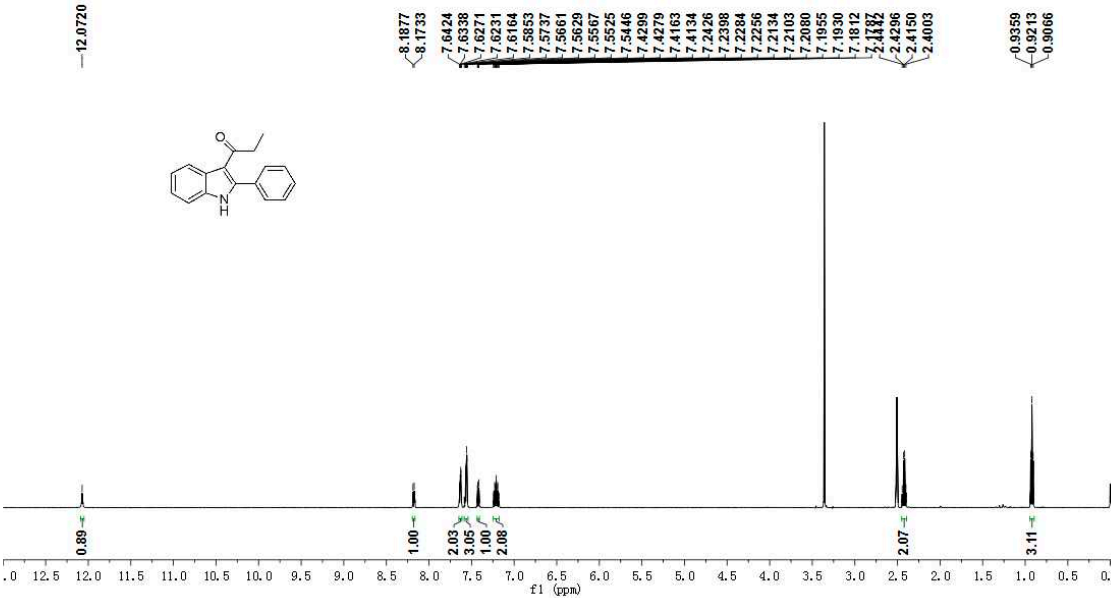

^{13}C NMR spectra (125 MHz, DMSO-d_6) of 3db

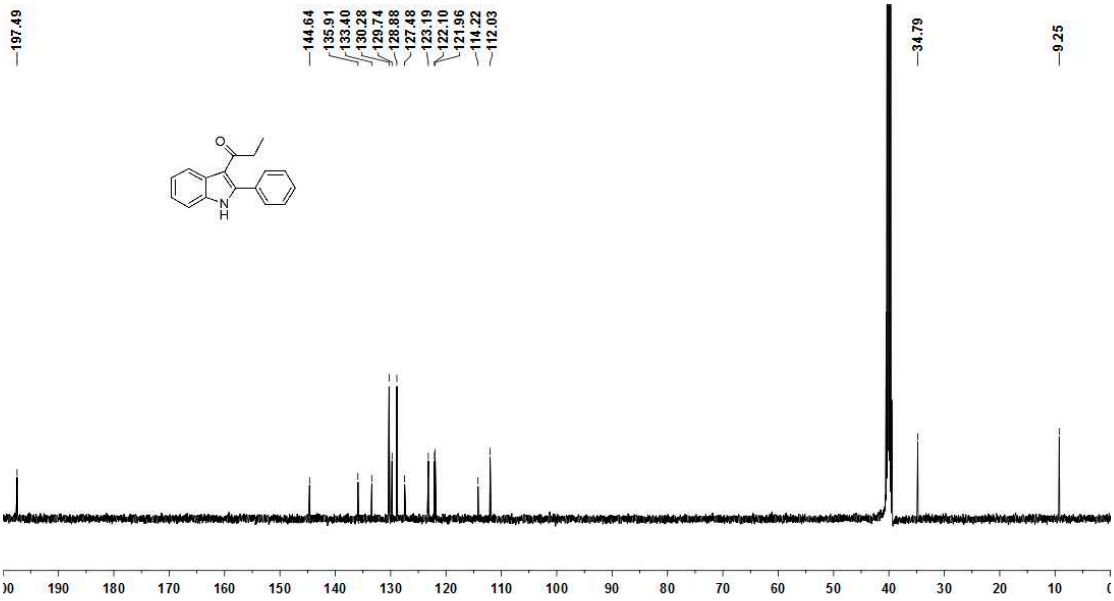

^{1}H NMR spectra (500 MHz, DMSO-d_6) of 3dc

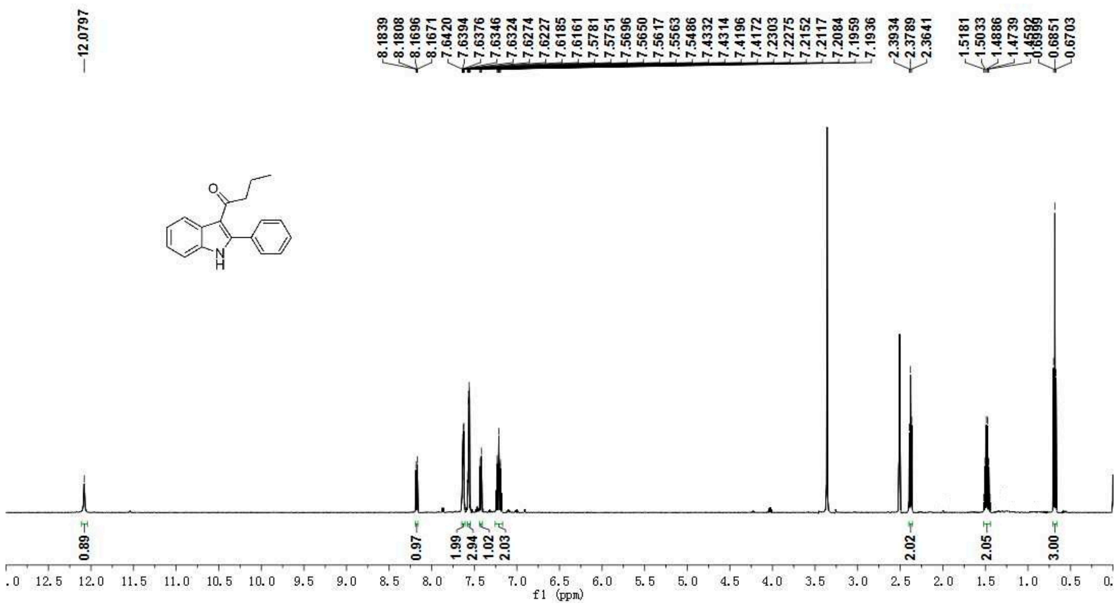

^{13}C NMR spectra (125 MHz, DMSO-d_6) of 3dc

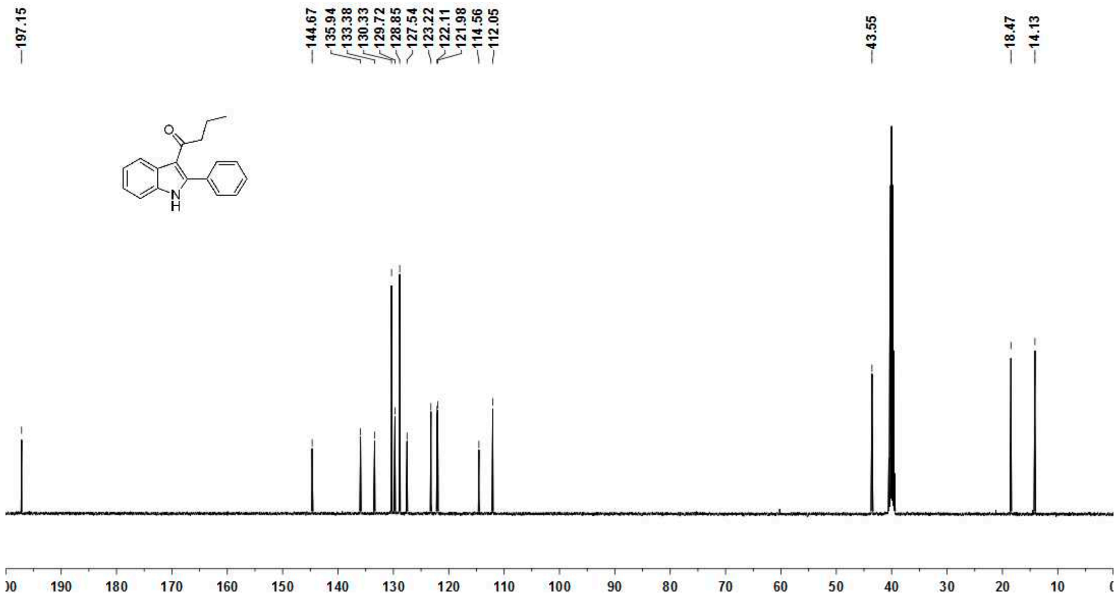

^{1}H NMR spectra (500 MHz, DMSO-d_6) of 3dd

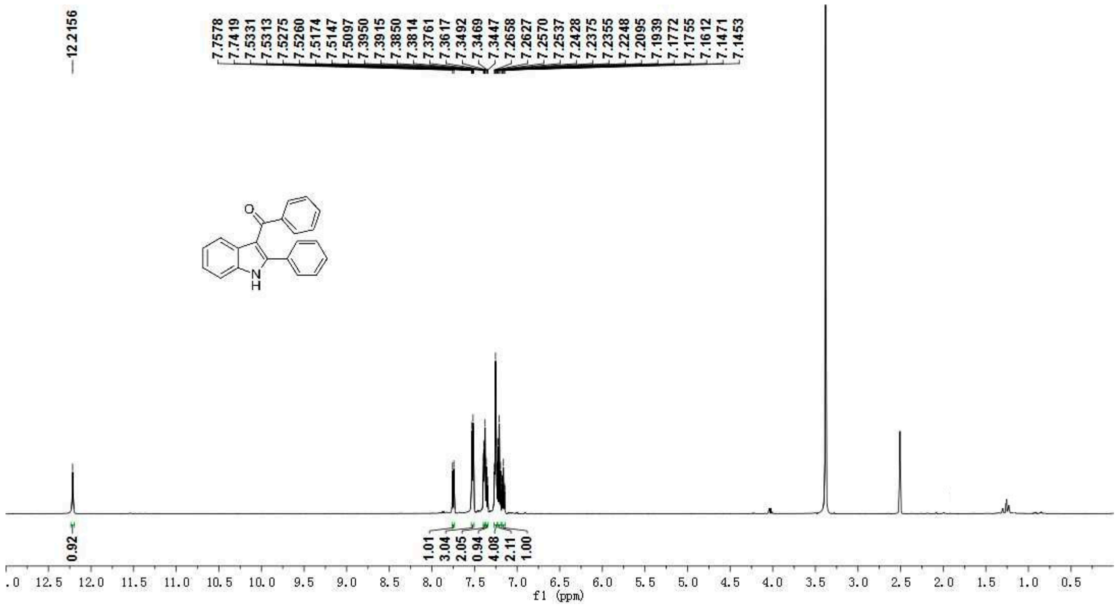

^{13}C NMR spectra (125 MHz, DMSO-d_6) of 3dd

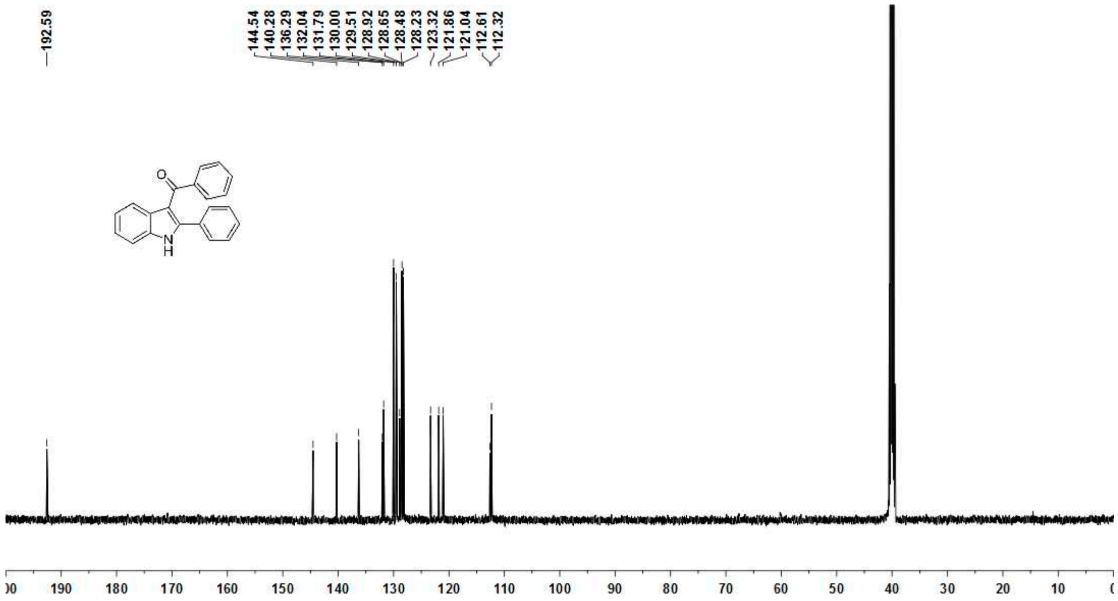

^{1}H NMR spectra (500 MHz, DMSO-d_6) of 3ea

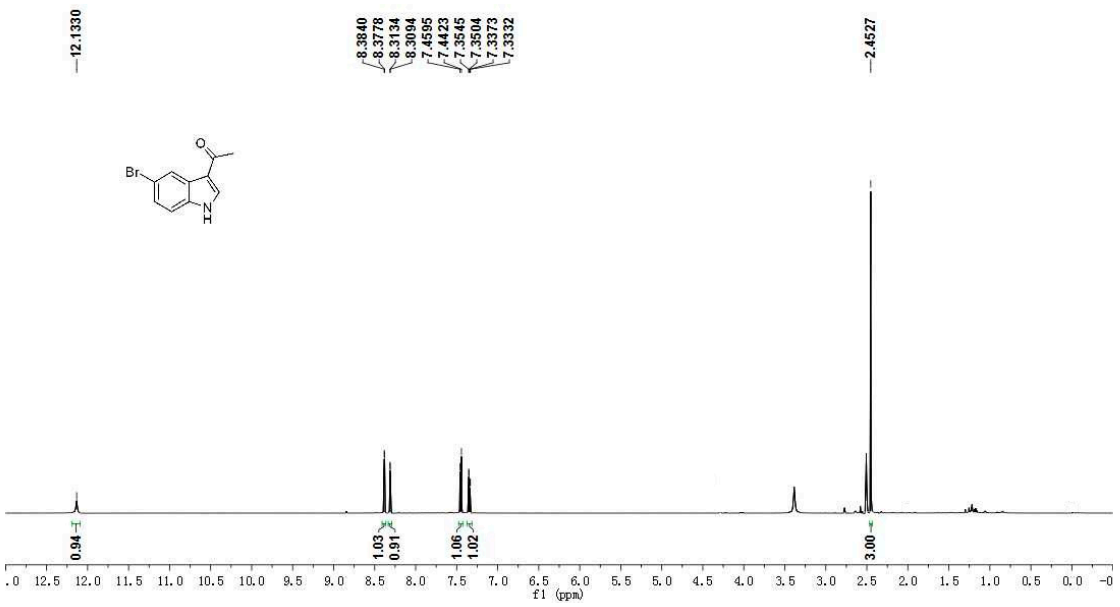

^{13}C NMR spectra (125 MHz, DMSO-d_6) of 3ea

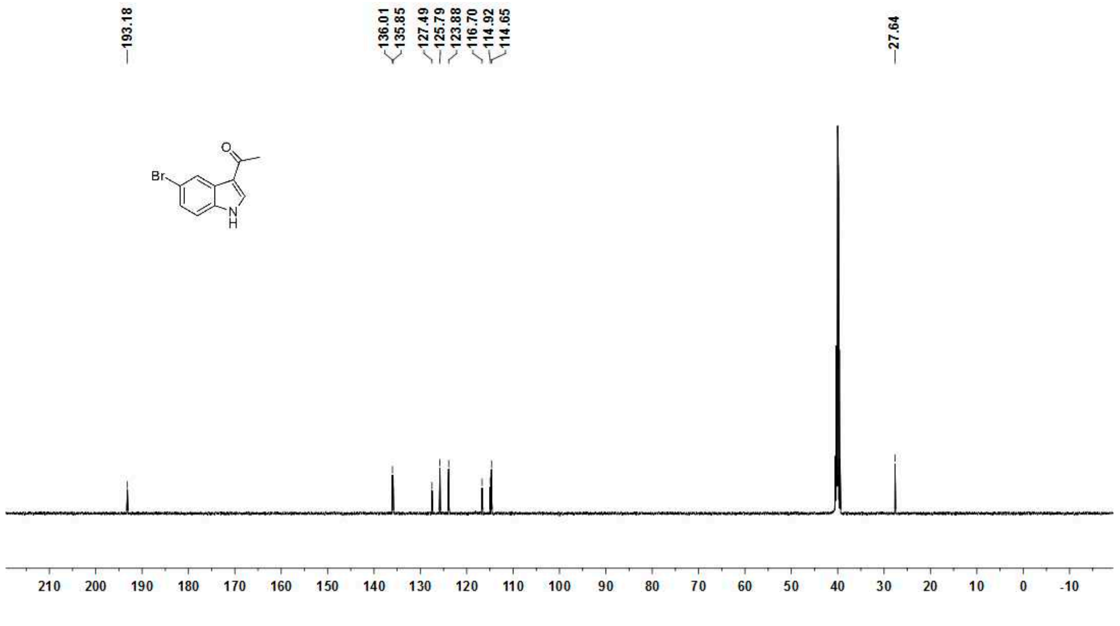

^{1}H NMR spectra (500 MHz, DMSO-*d*6) of 3eb

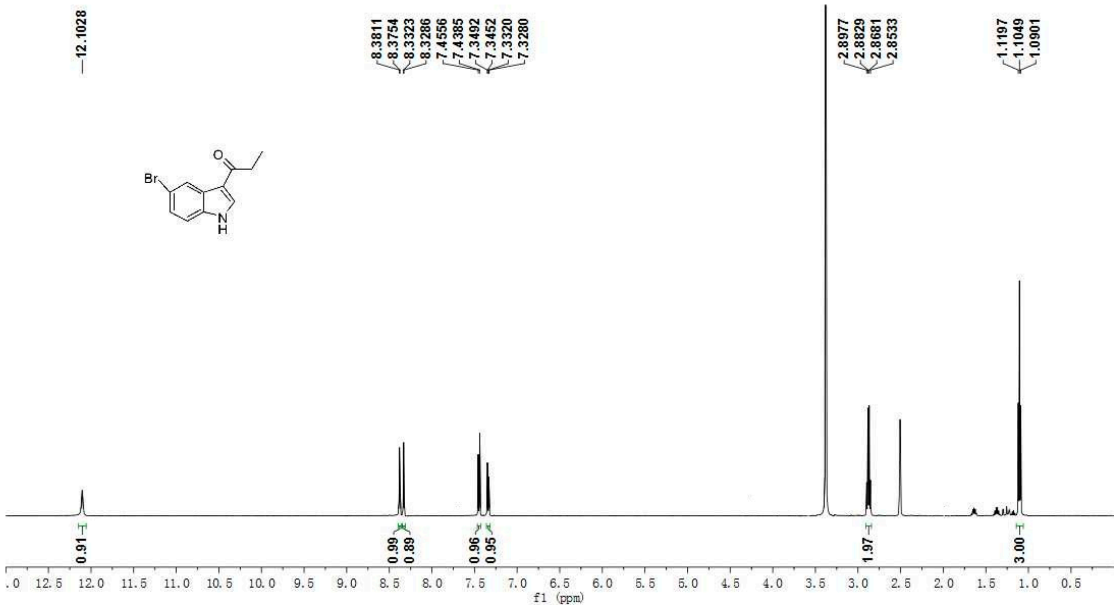

^{13}C NMR spectra (125 MHz, DMSO-*d*6) of 3eb

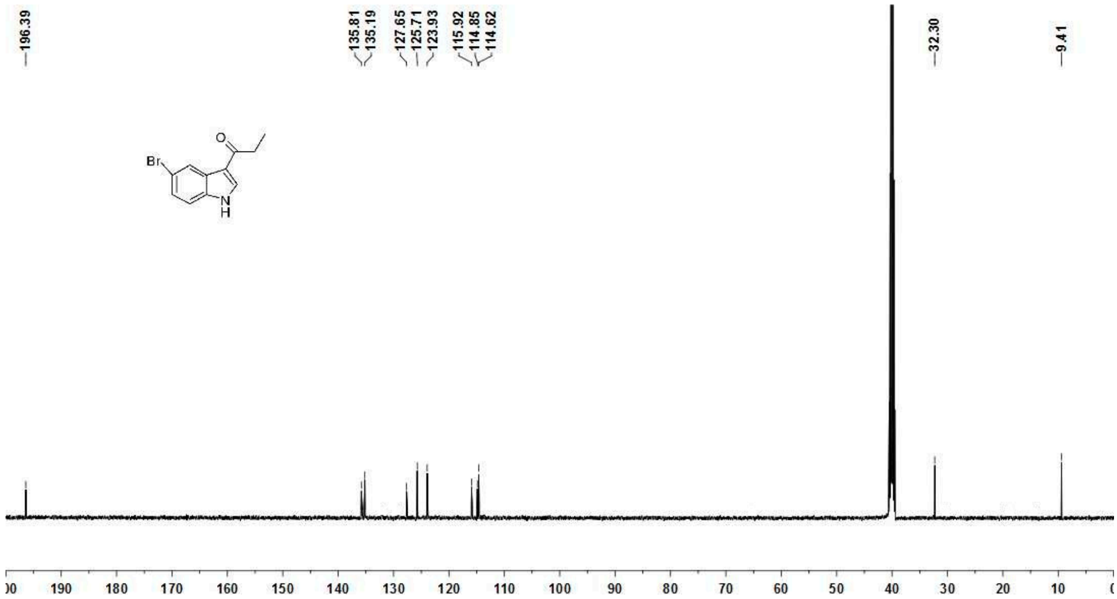

^{1}H NMR spectra (500 MHz, DMSO-d_6) of 3ec

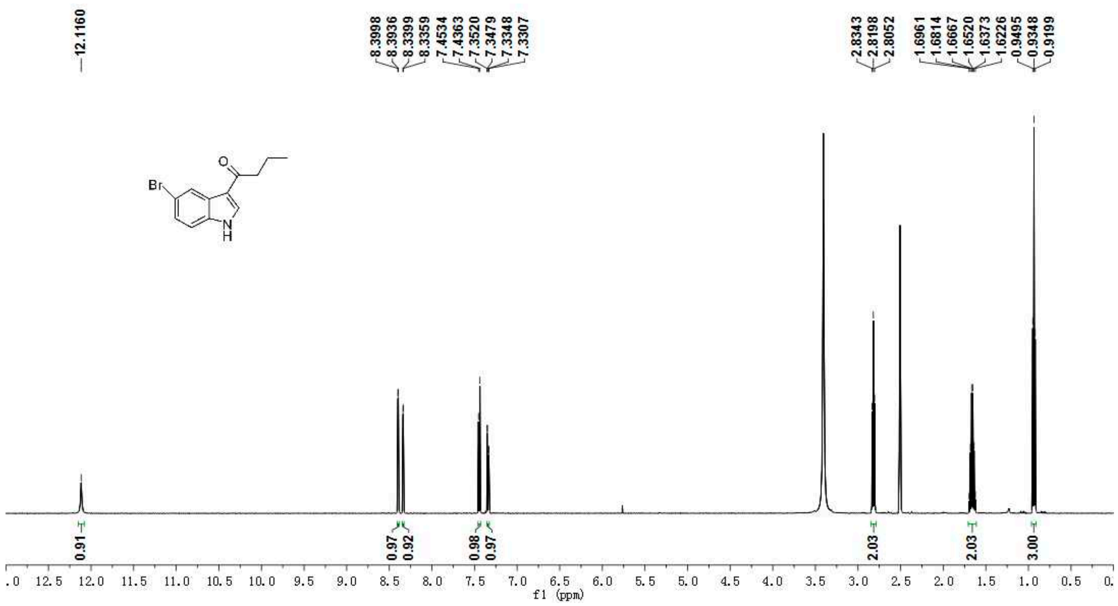

^{13}C NMR spectra (125 MHz, DMSO-d_6) of 3ec

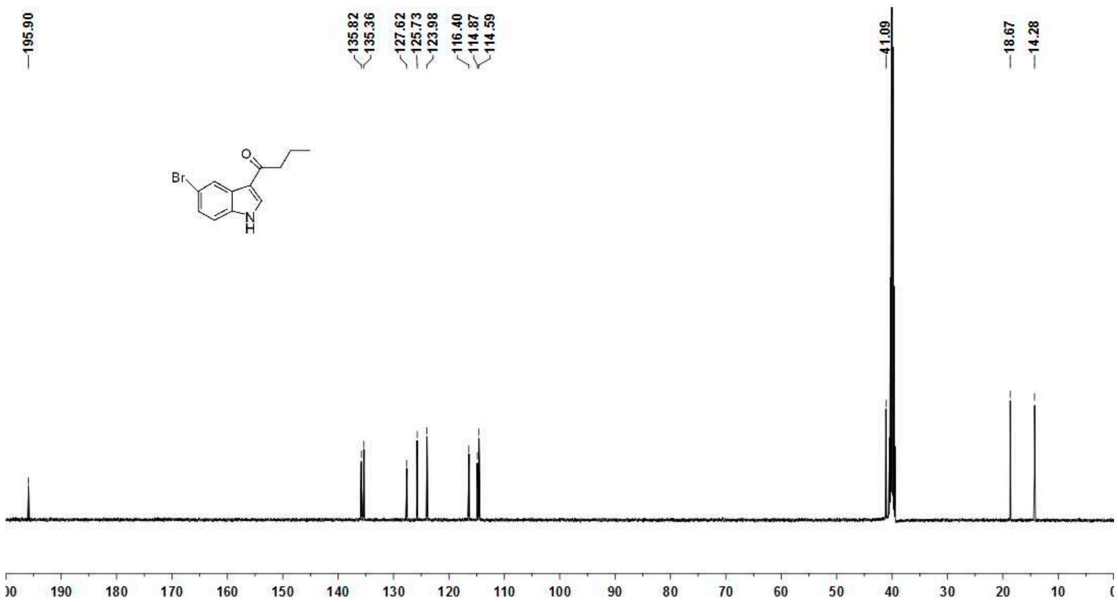

^{1}H NMR spectra (500 MHz, DMSO-d_6) of 3ed

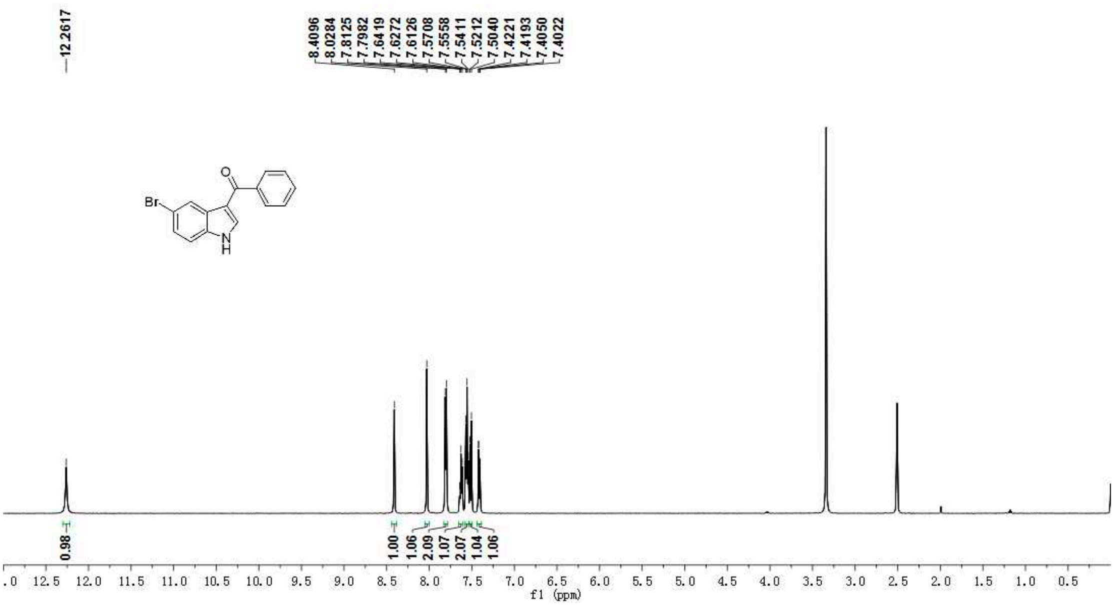

^{13}C NMR spectra (125 MHz, DMSO-d_6) of 3ed

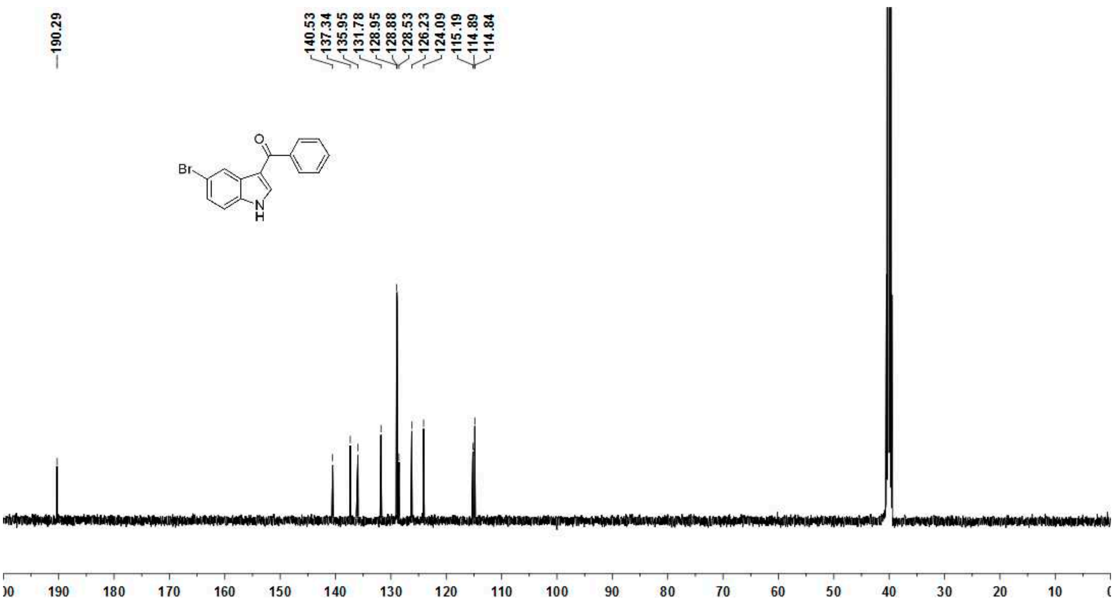

^{1}H NMR spectra (500 MHz, DMSO-d_6) of 3fa

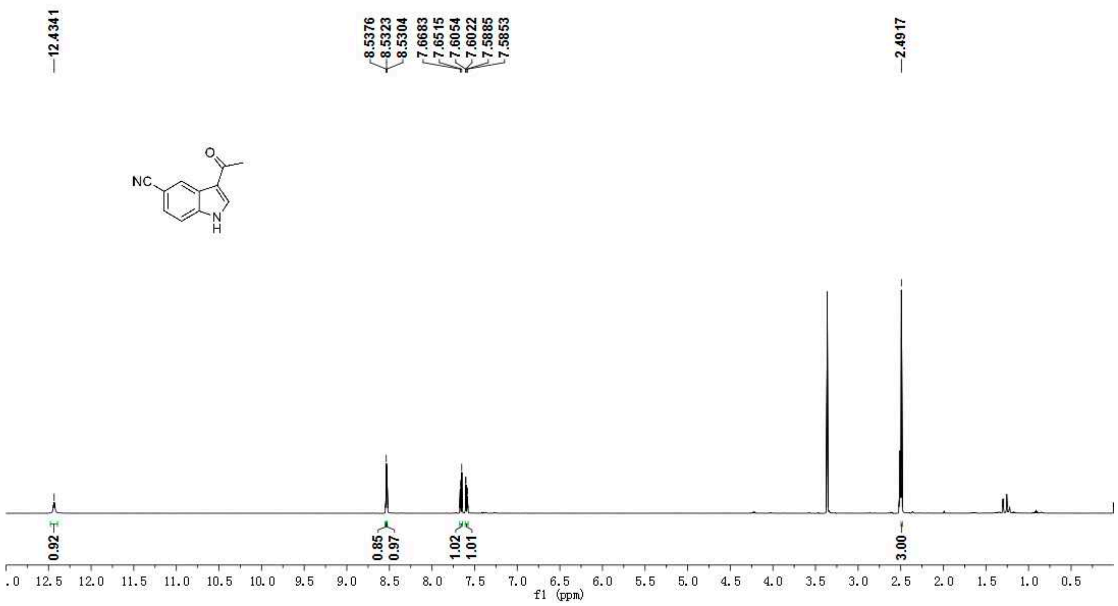

^{13}C NMR spectra (125 MHz, DMSO-d_6) of 3fa

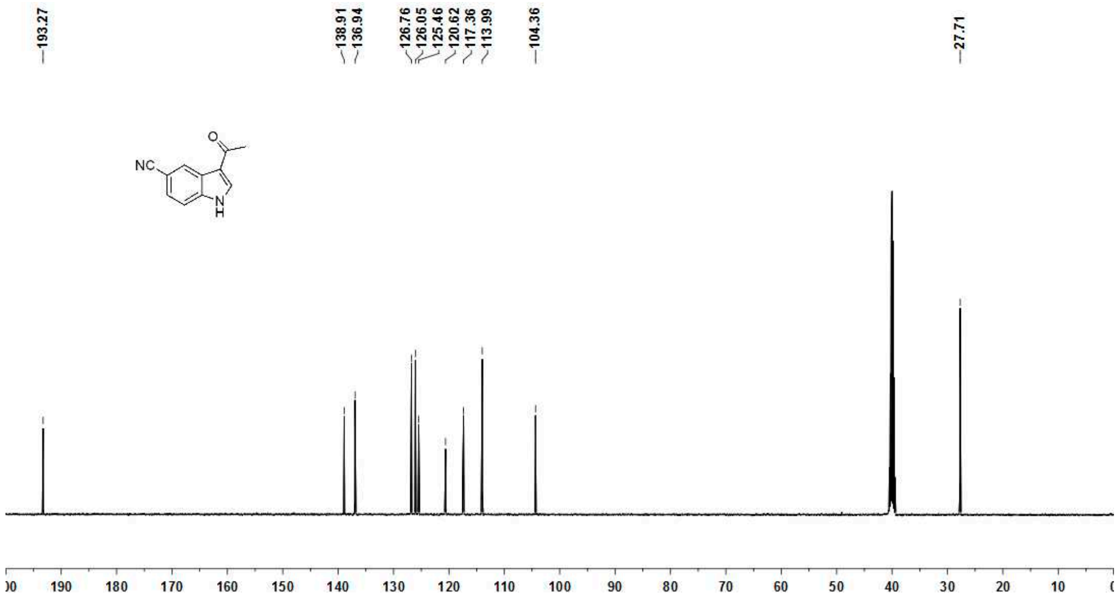

^{1}H NMR spectra (500 MHz, DMSO-d_6) of 3fb

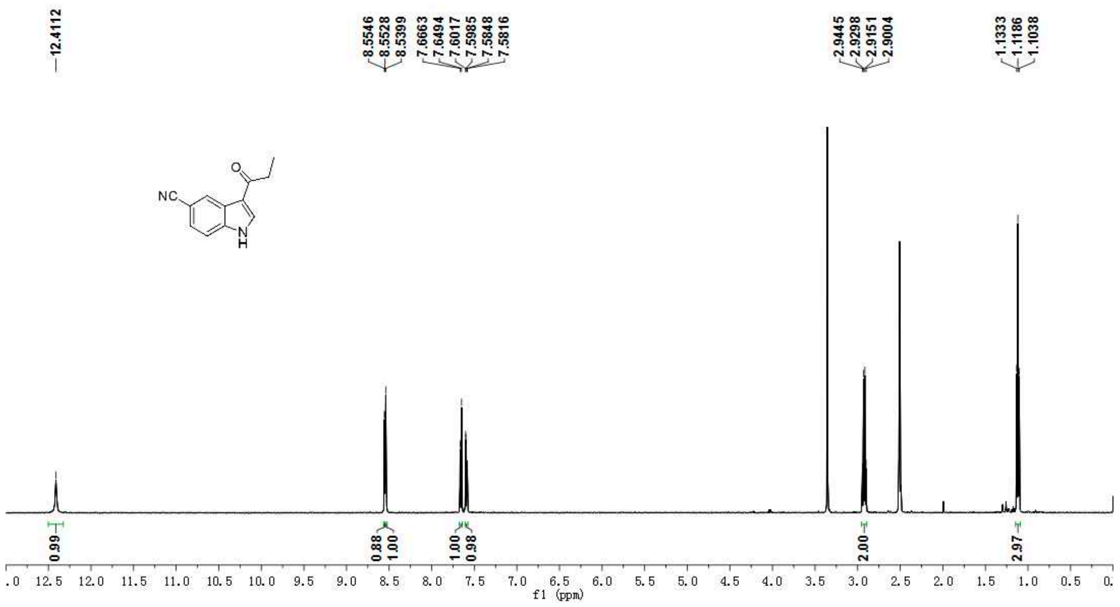

^{13}C NMR spectra (125 MHz, DMSO-d_6) of 3fb

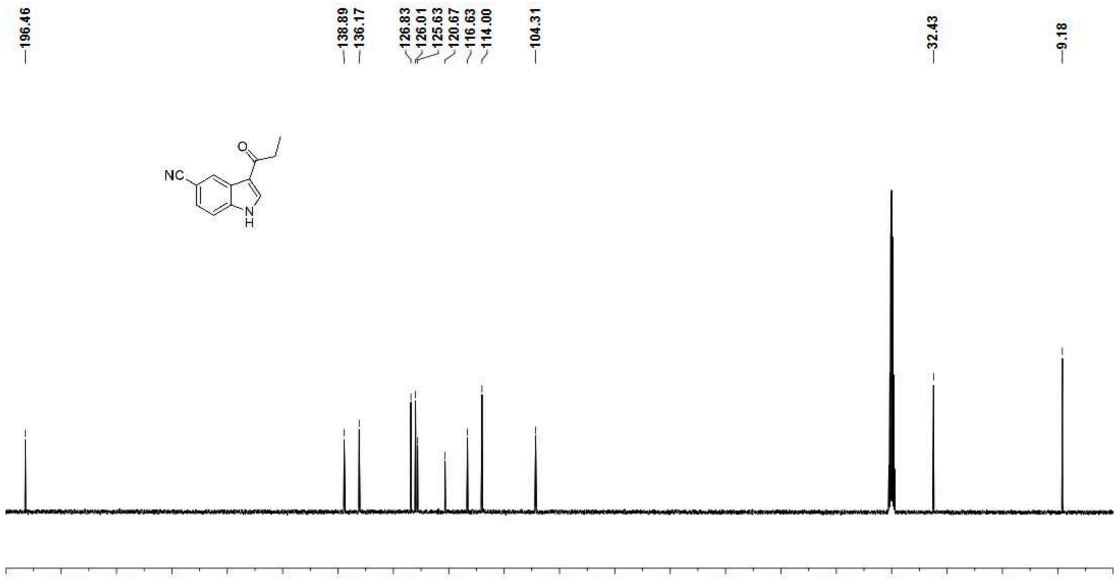

^{1}H NMR spectra (500 MHz, DMSO-d_6) of 3fc

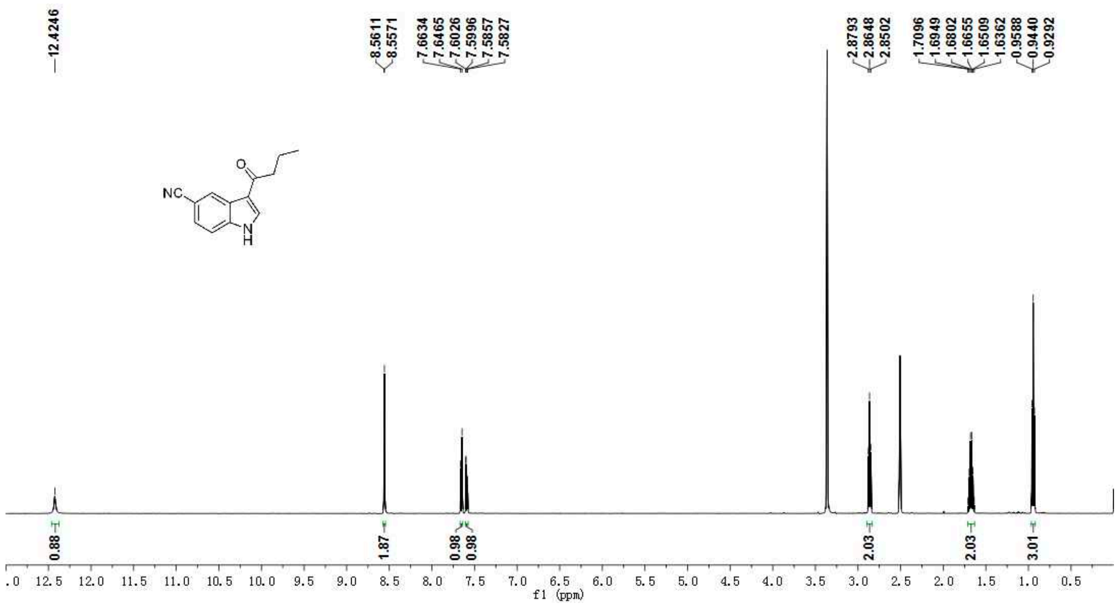

^{13}C NMR spectra (125 MHz, DMSO-d_6) of 3fc

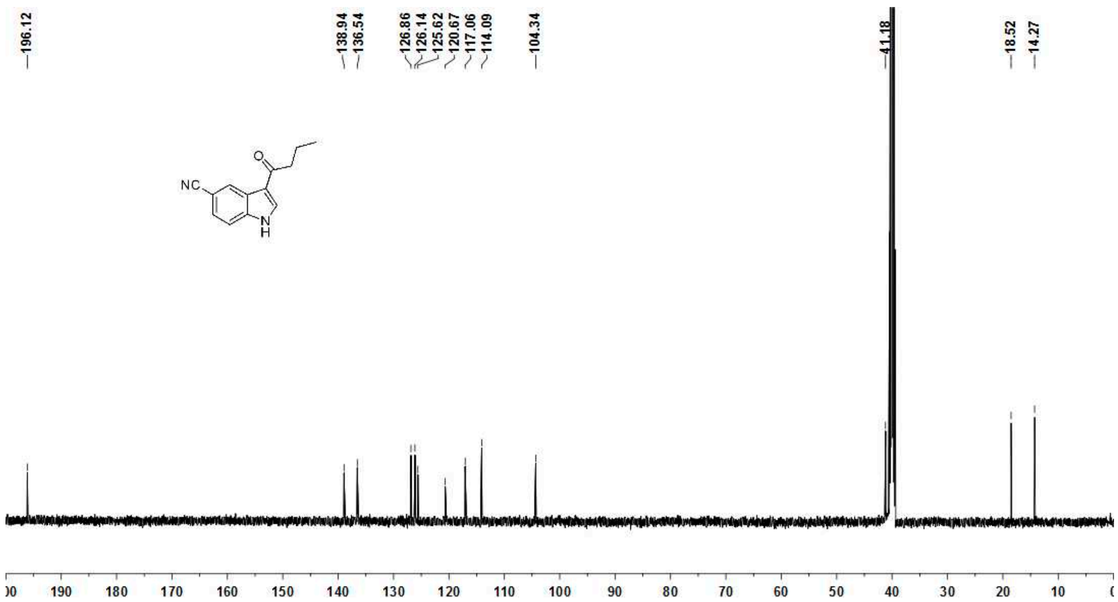

^{1}H NMR spectra (500 MHz, DMSO-d_6) of 3fd

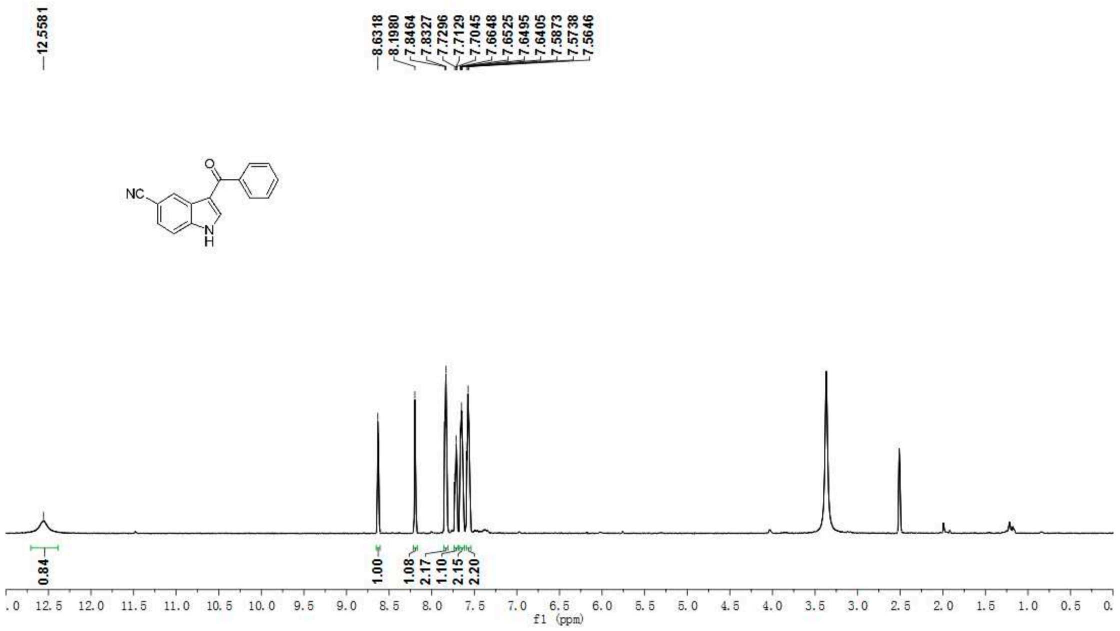

^{13}C NMR spectra (125 MHz, DMSO-d_6) of 3fd

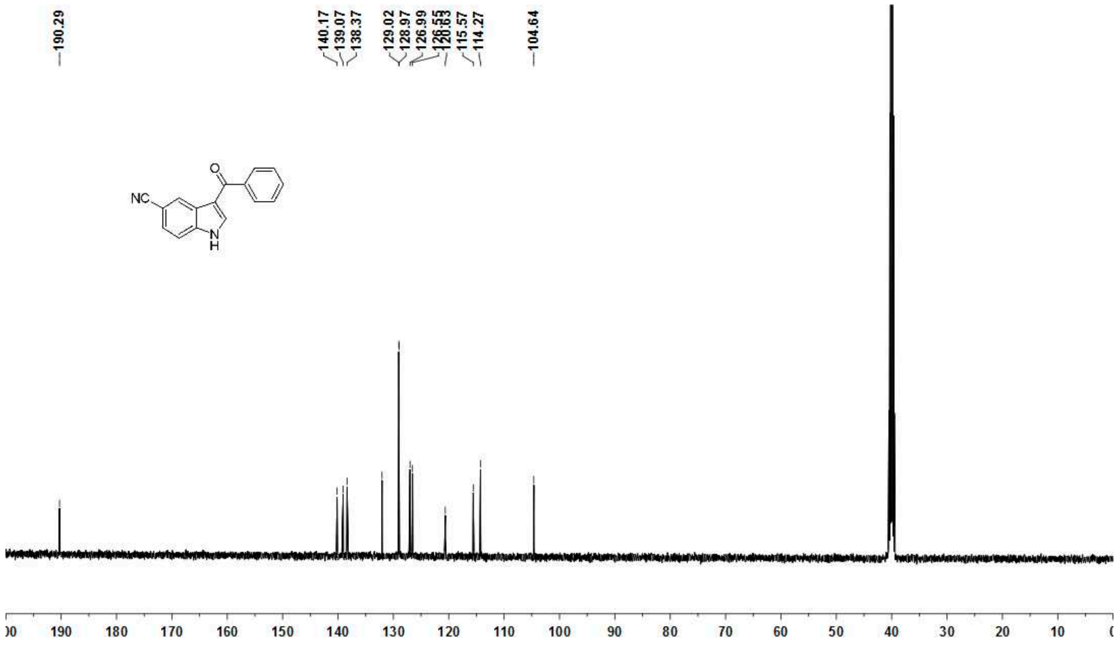

^{1}H NMR spectra (500 MHz, DMSO-d_6) of 3ga

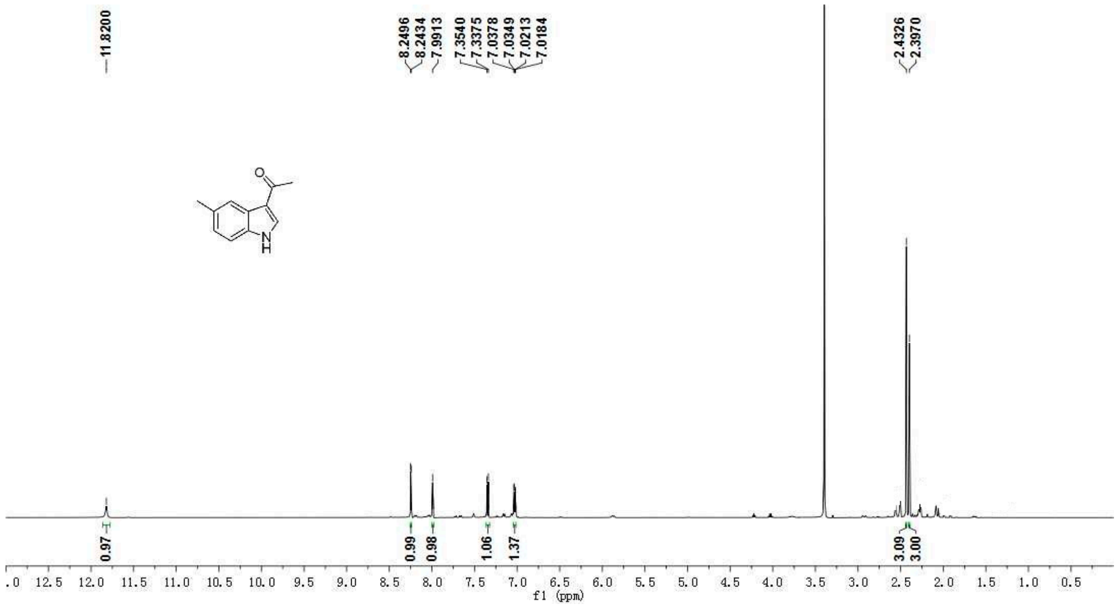

^{13}C NMR spectra (125 MHz, DMSO-d_6) of 3ga

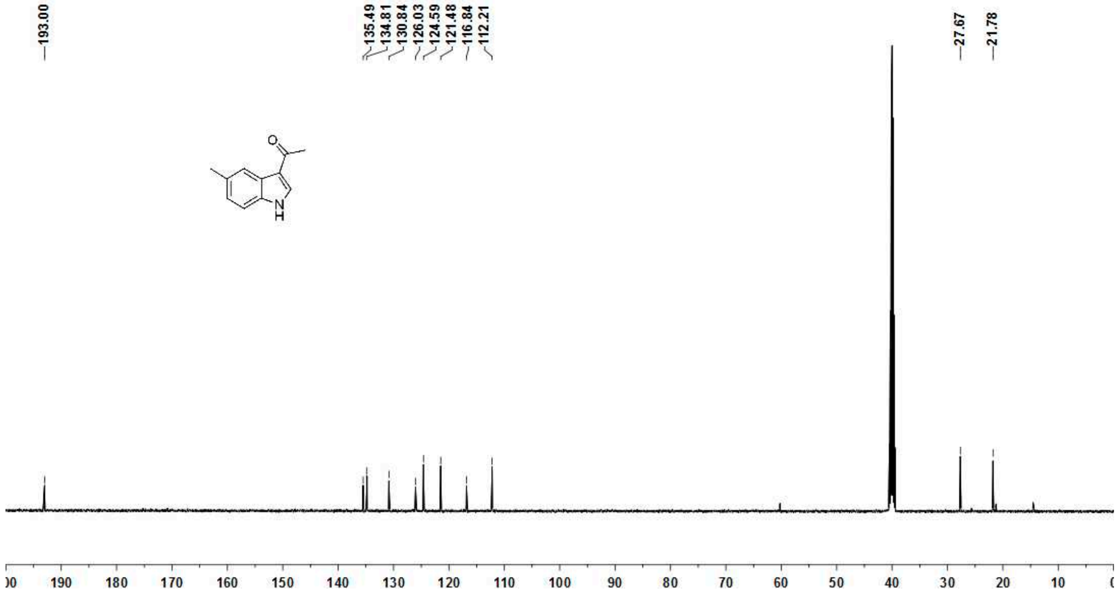

^{1}H NMR spectra (500 MHz, DMSO-*d*6) of 3gb

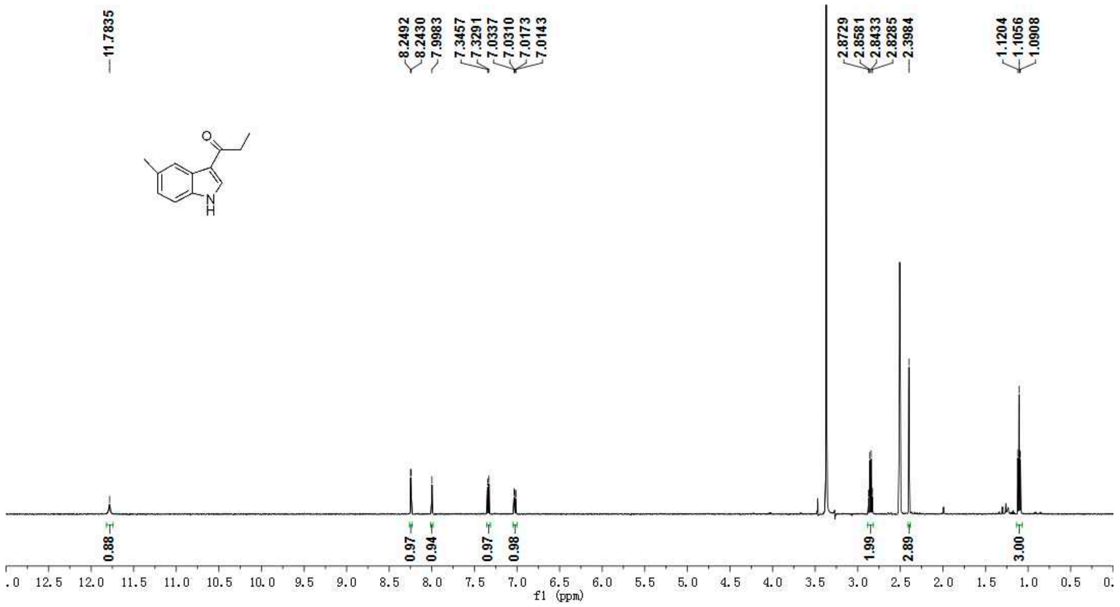

^{13}C NMR spectra (125 MHz, DMSO-*d*6) of 3gb

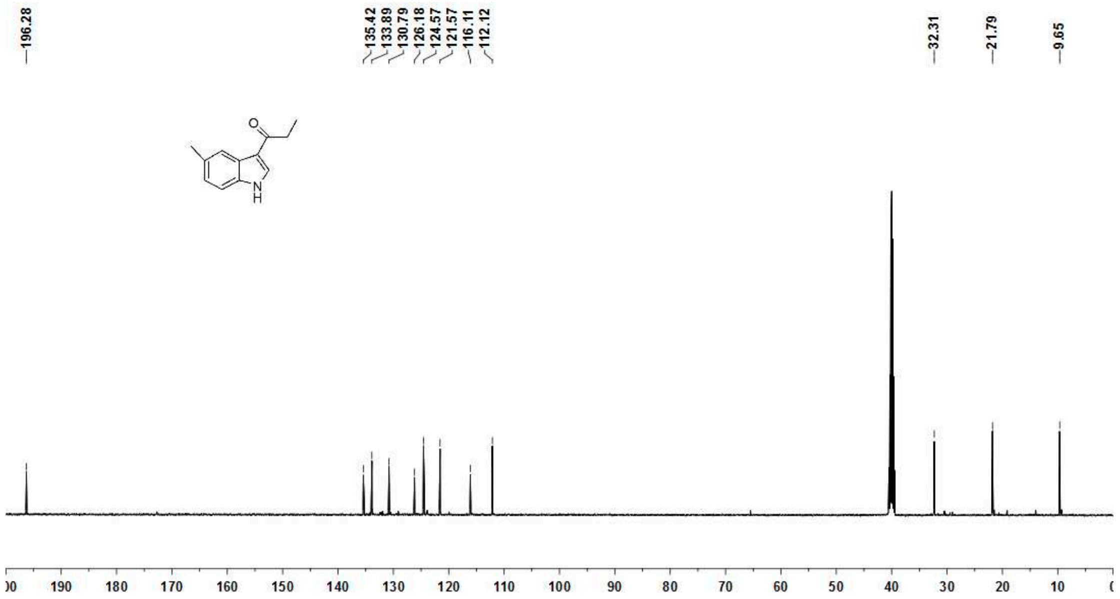

^{1}H NMR spectra (500 MHz, DMSO-d_6) of 3gc

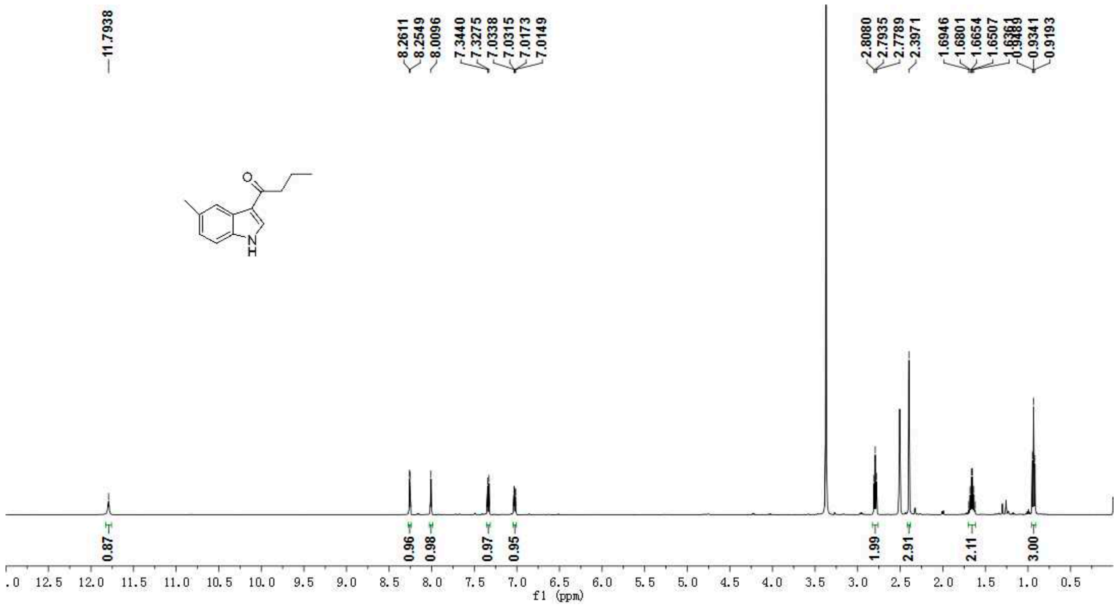

^{13}C NMR spectra (125 MHz, DMSO-d_6) of 3gc

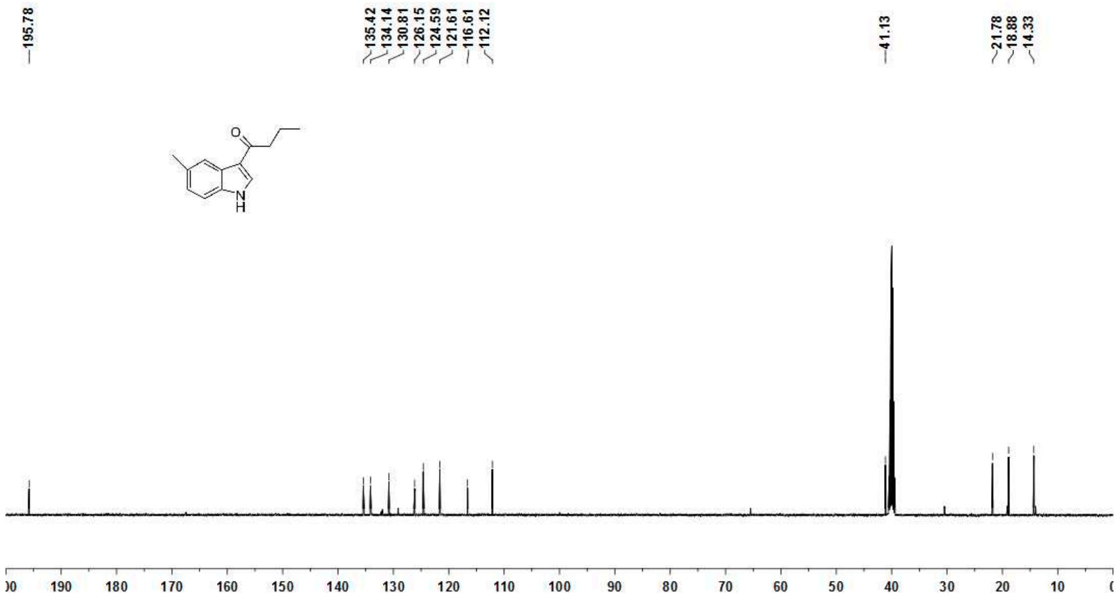

^{1}H NMR spectra (500 MHz, DMSO-d_6) of 3gd

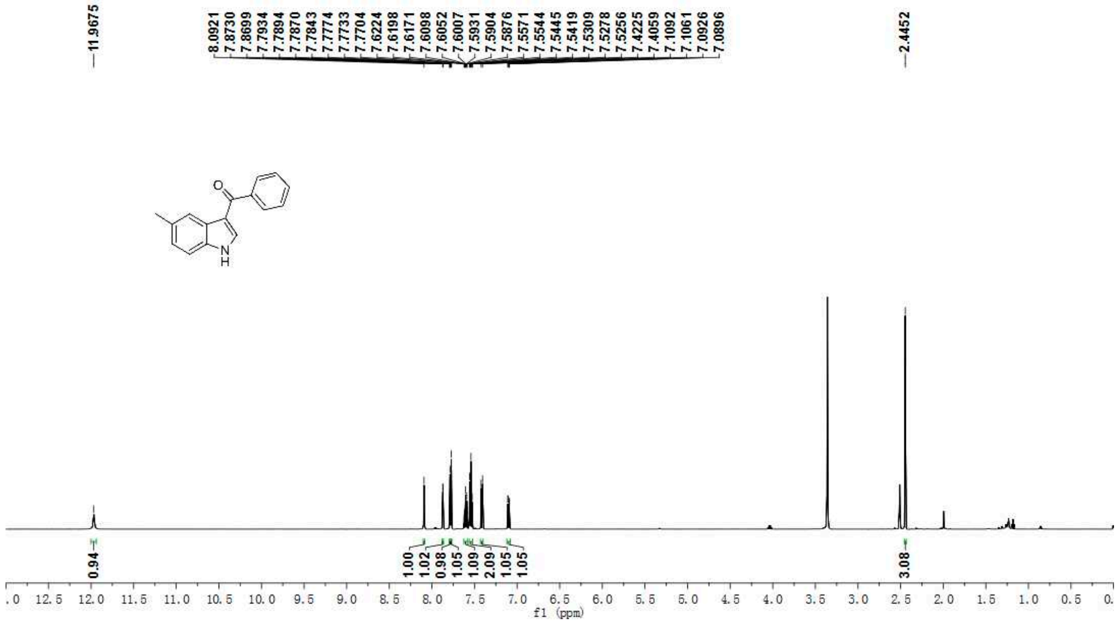

^{13}C NMR spectra (125 MHz, DMSO-d_6) of 3gd

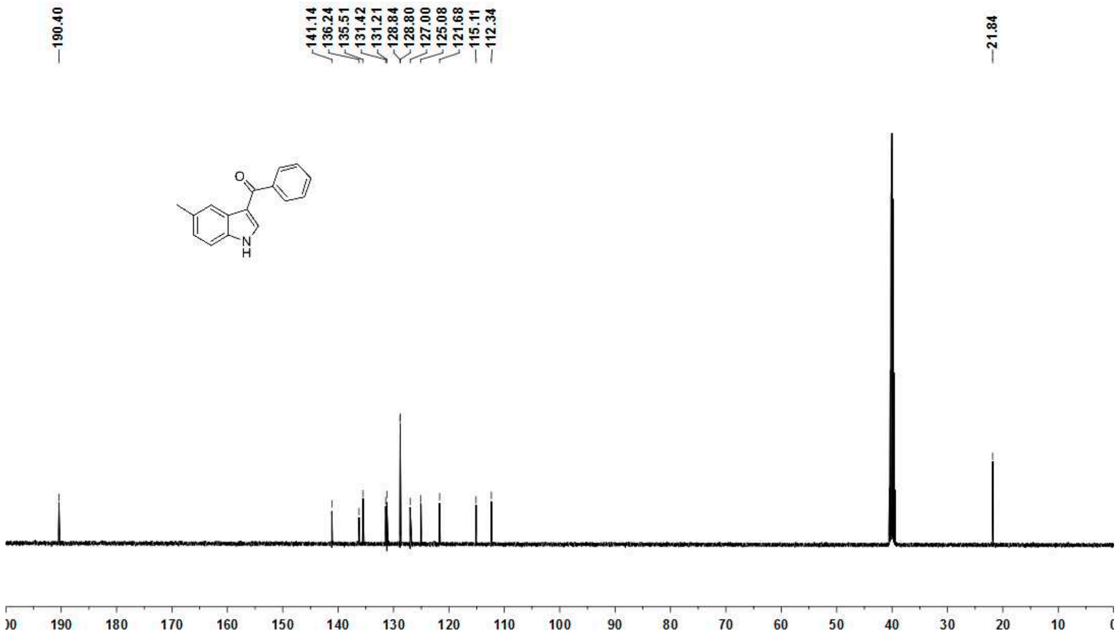

^{1}H NMR spectra (500 MHz, DMSO-d_6) of 3ha

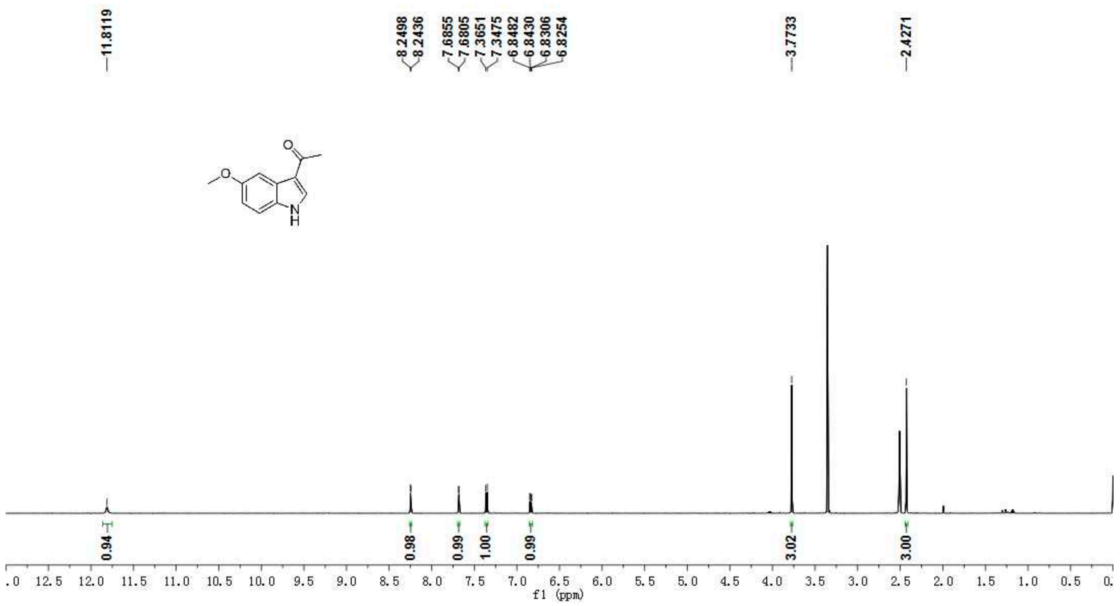

^{13}C NMR spectra (125 MHz, DMSO-d_6) of 3ha

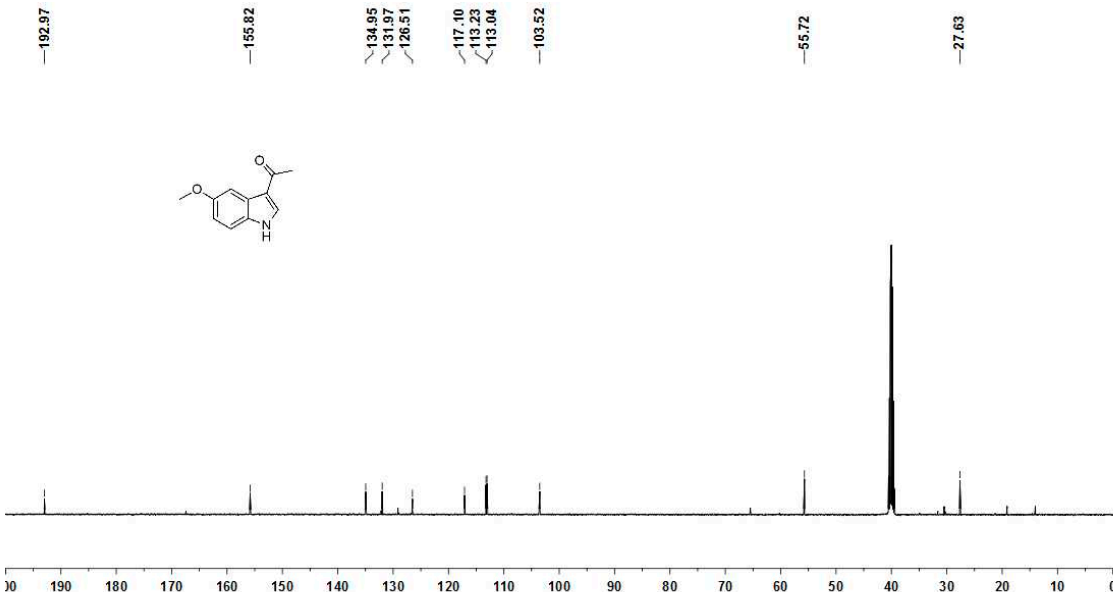

^{1}H NMR spectra (500 MHz, DMSO-d_6) of 3hb

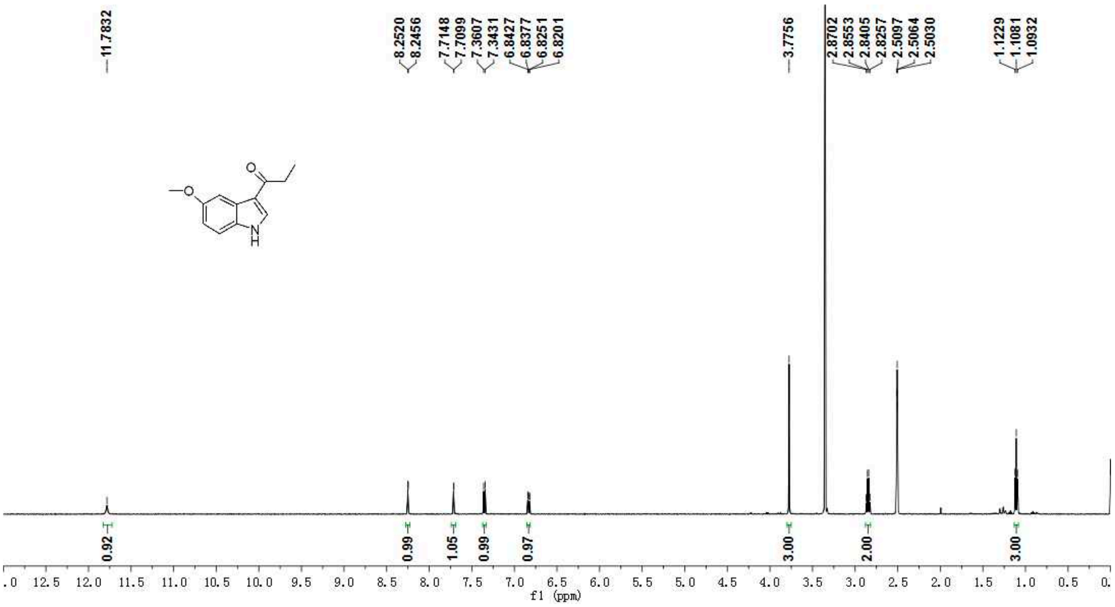

^{13}C NMR spectra (125 MHz, DMSO-d_6) of 3hb

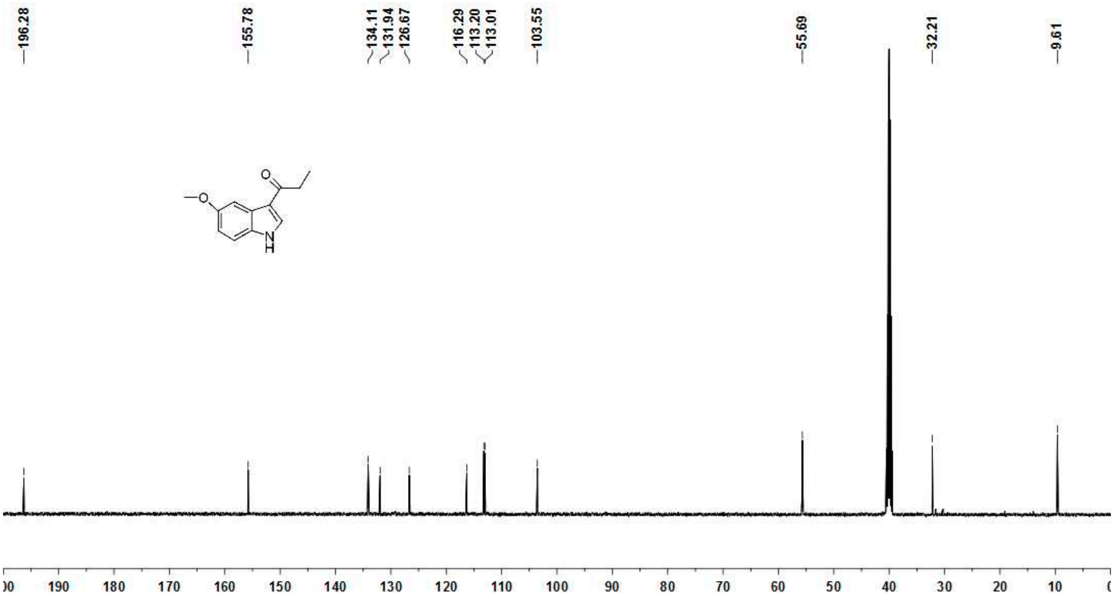

^{1}H NMR spectra (500 MHz, DMSO-d_6) of 3hc

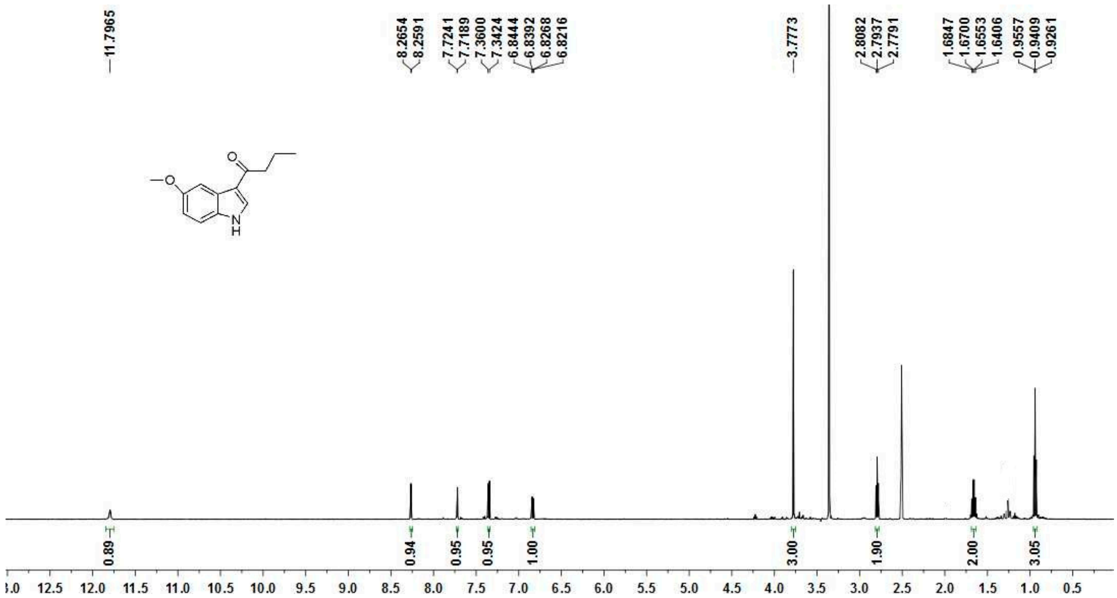

^{13}C NMR spectra (125 MHz, DMSO-d_6) of 3hc

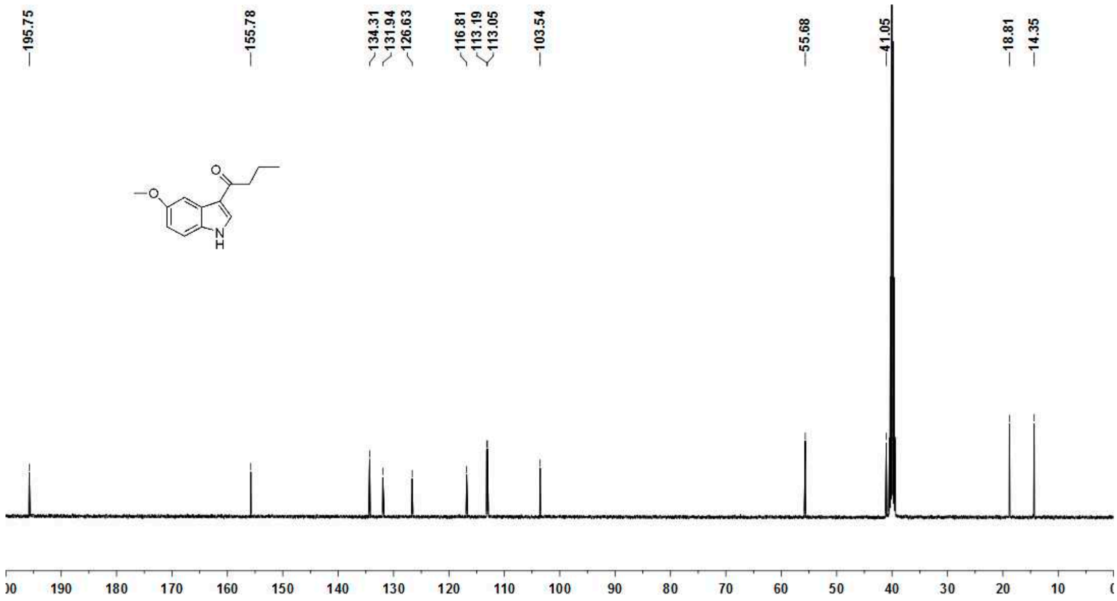

^{1}H NMR spectra (500 MHz, DMSO-d_6) of 3hd

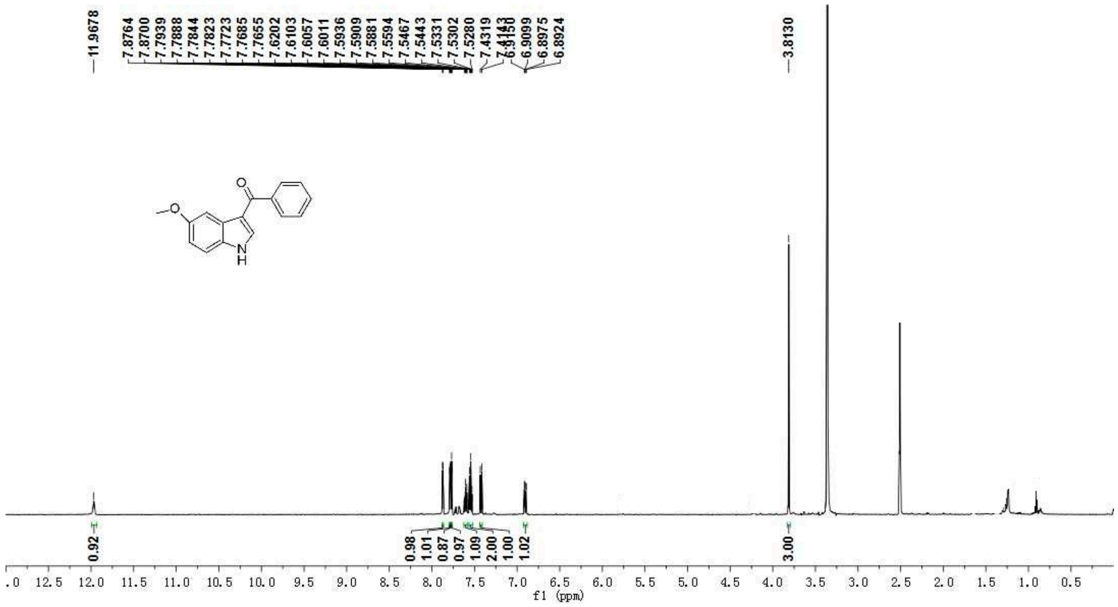

^{13}C NMR spectra (125 MHz, DMSO-d_6) of 3hd

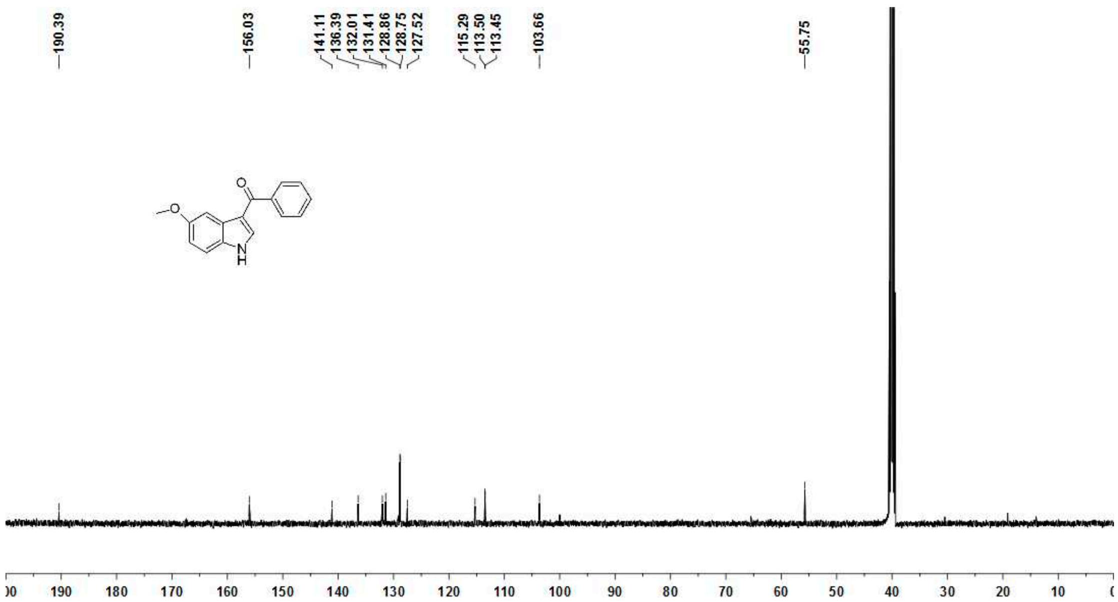

^{1}H NMR spectra (500 MHz, DMSO-d_6) of 3ia

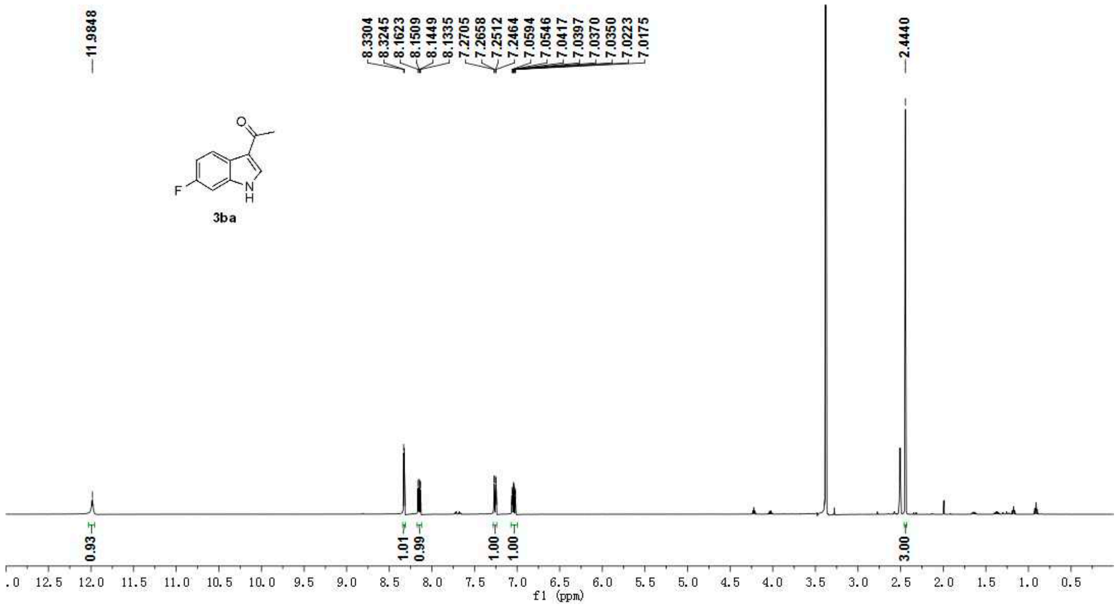

^{13}C NMR spectra (125 MHz, DMSO-d_6) of 3ia

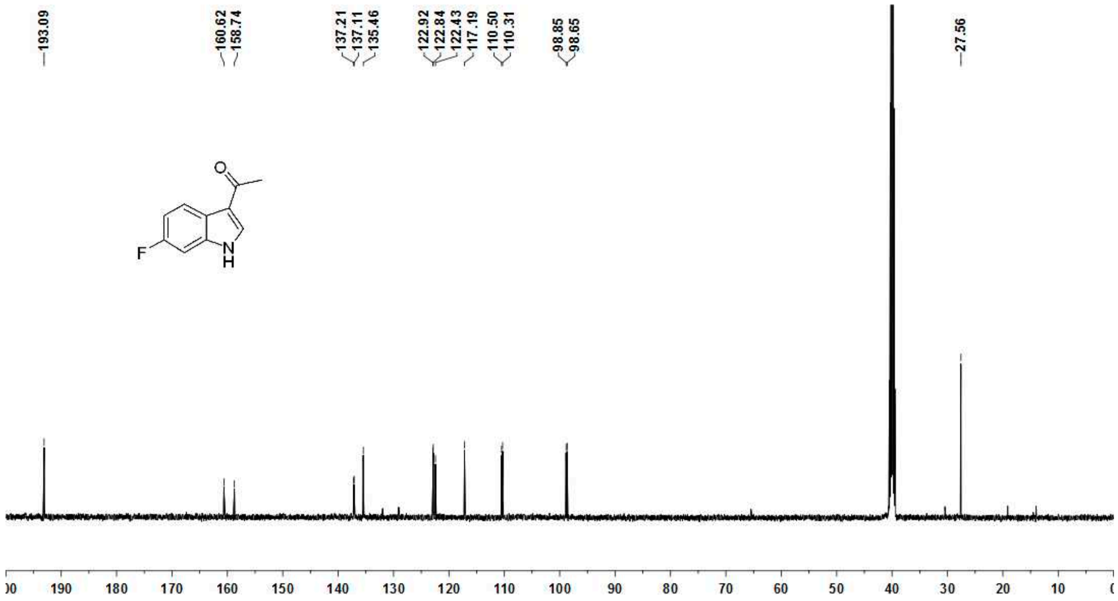

^{1}H NMR spectra (500 MHz, DMSO-*d*[6]) of 3ib

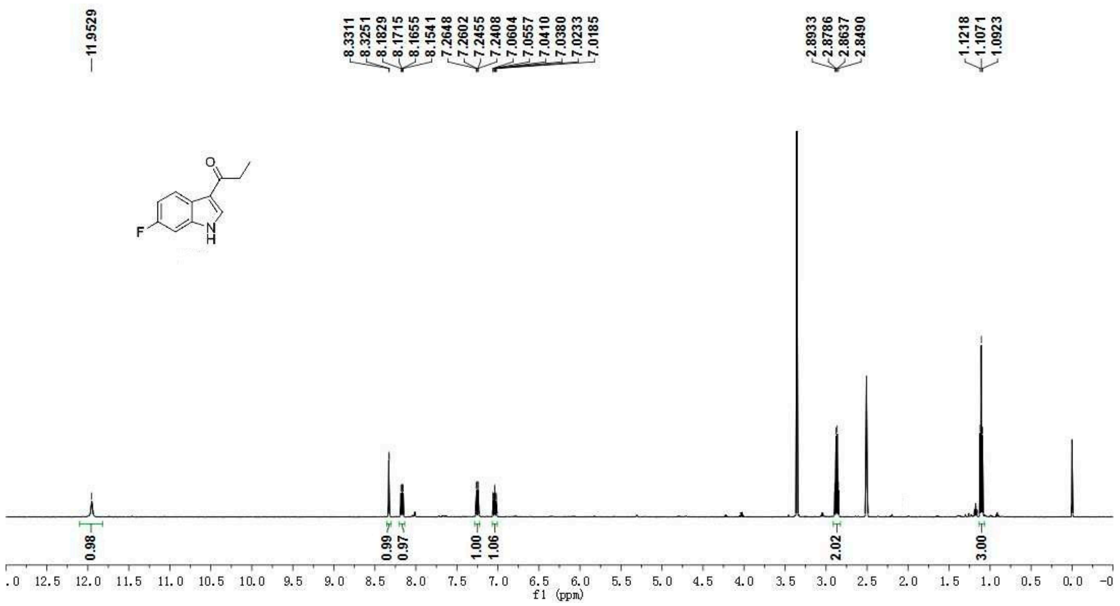

^{13}C NMR spectra (125 MHz, DMSO-*d*[6]) of 3ib

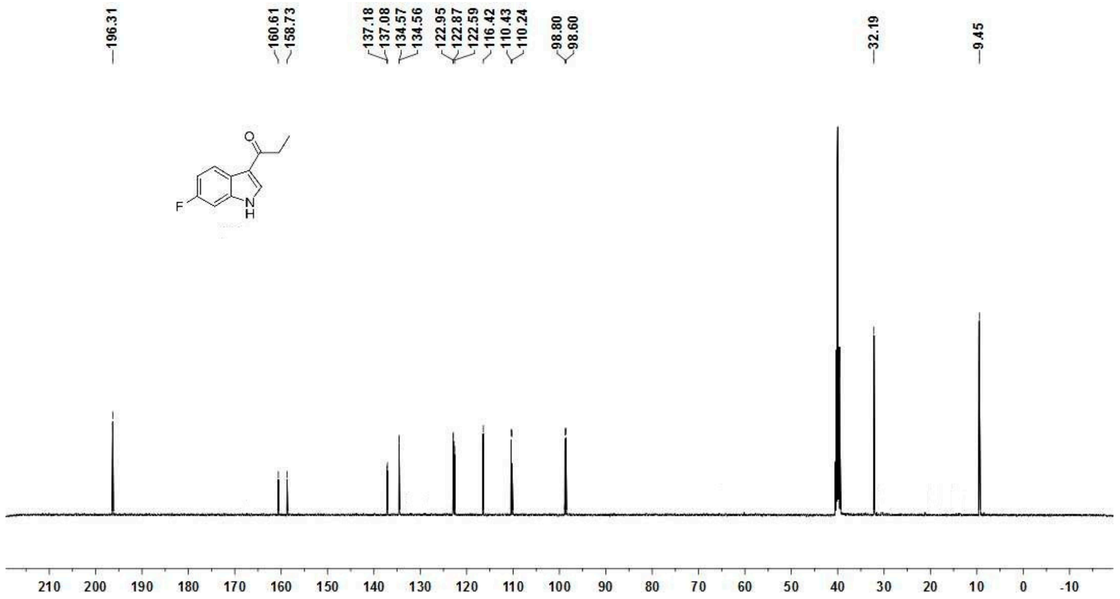

^{1}H NMR spectra (500 MHz, DMSO-d_6) of 3ic

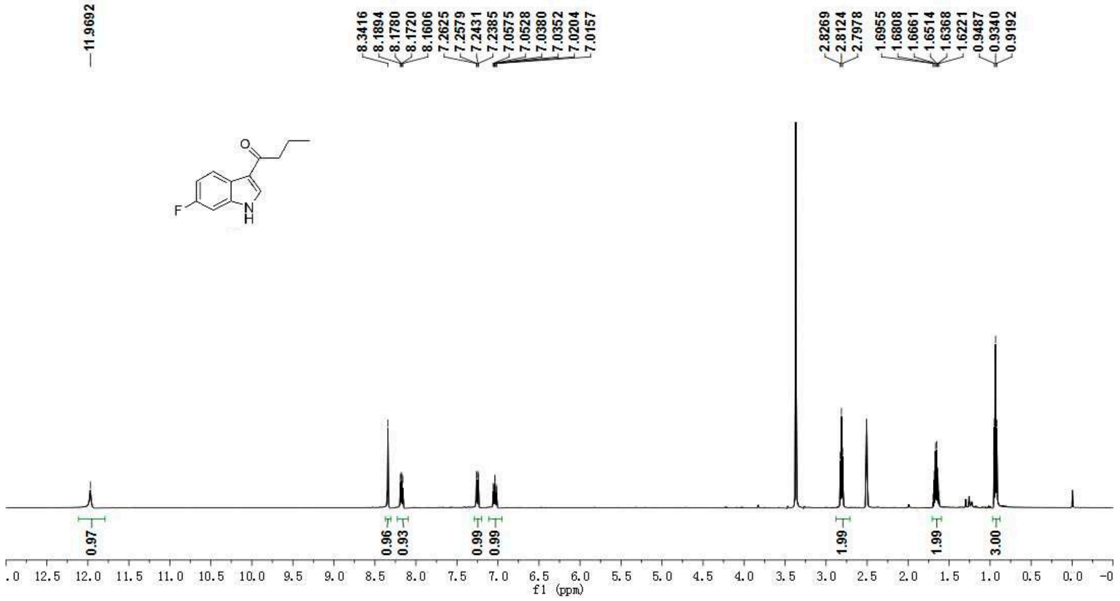

^{13}C NMR spectra (125 MHz, DMSO-d_6) of 3ic

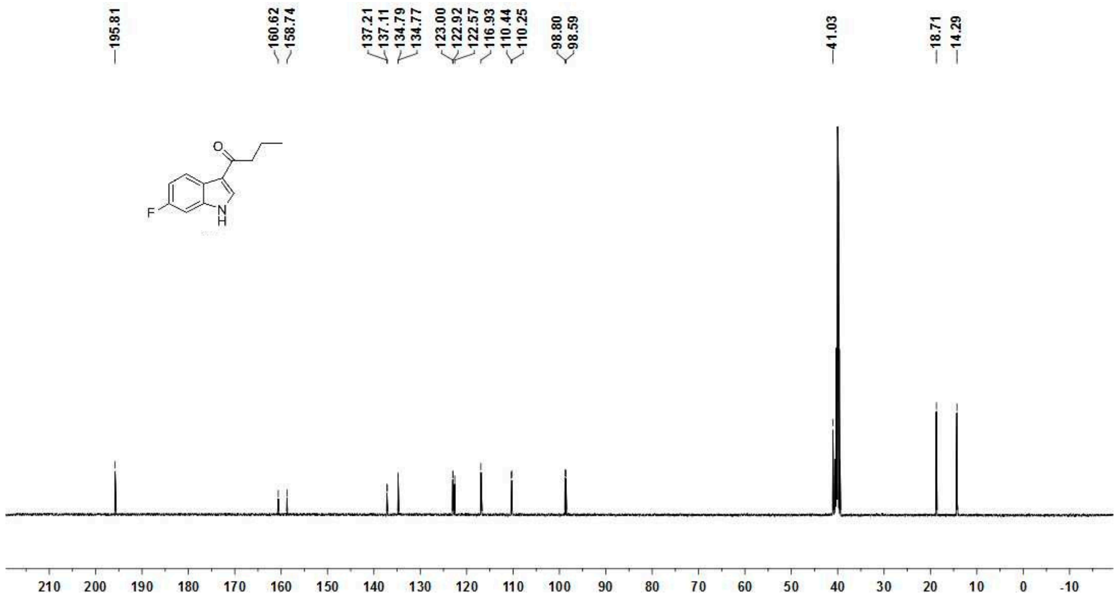

^{1}H NMR spectra (500 MHz, DMSO-d_6) of 3id

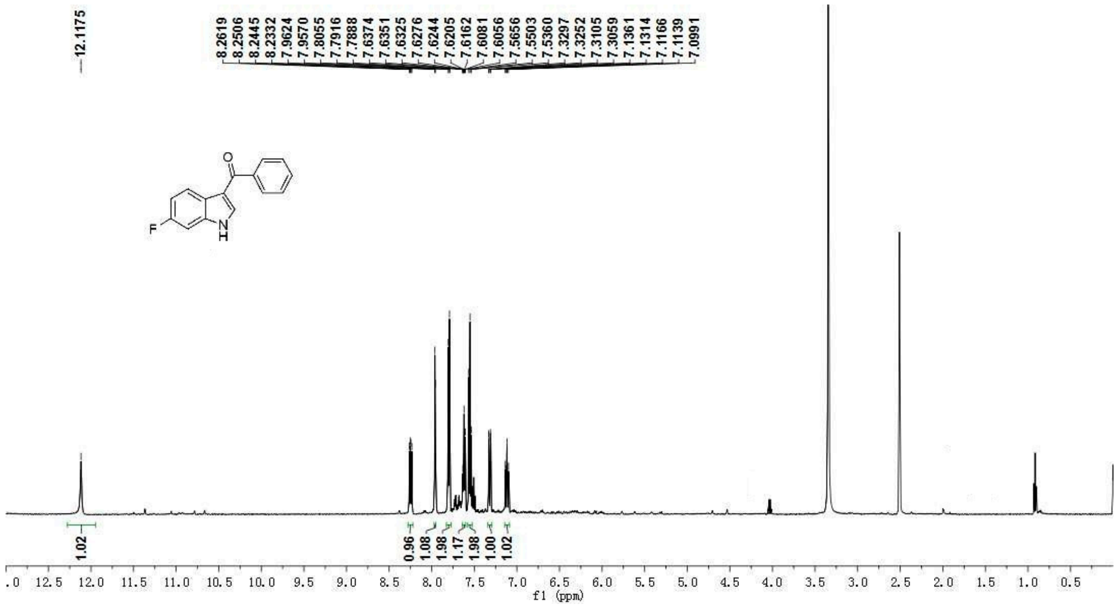

^{13}C NMR spectra (125 MHz, DMSO-d_6) of 3id

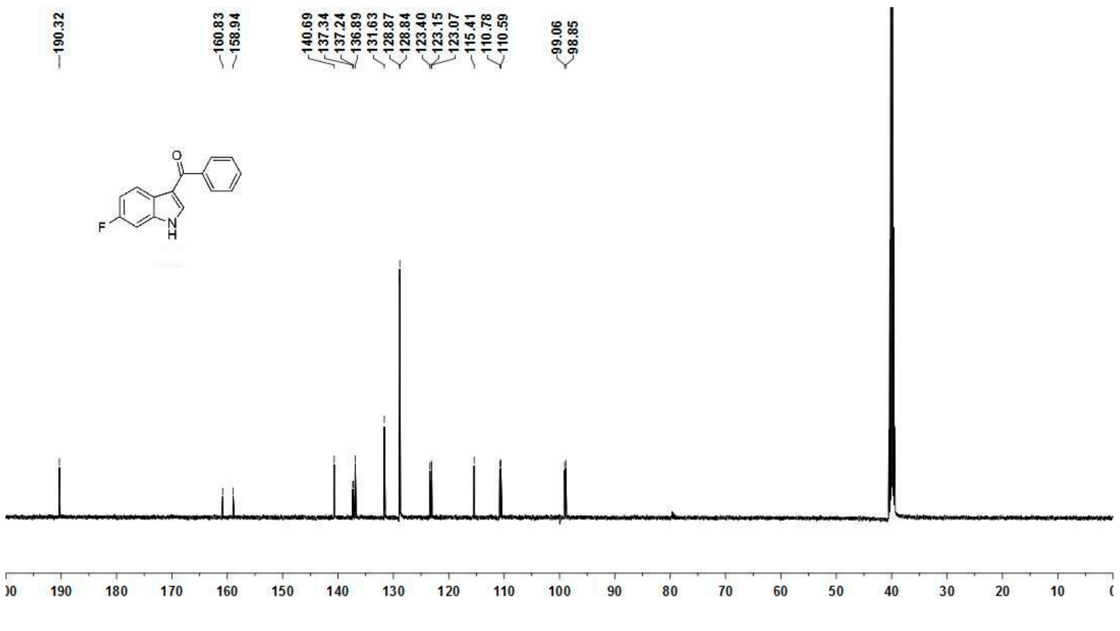

^{1}H NMR spectra (500 MHz, DMSO-d_6) of 3ja

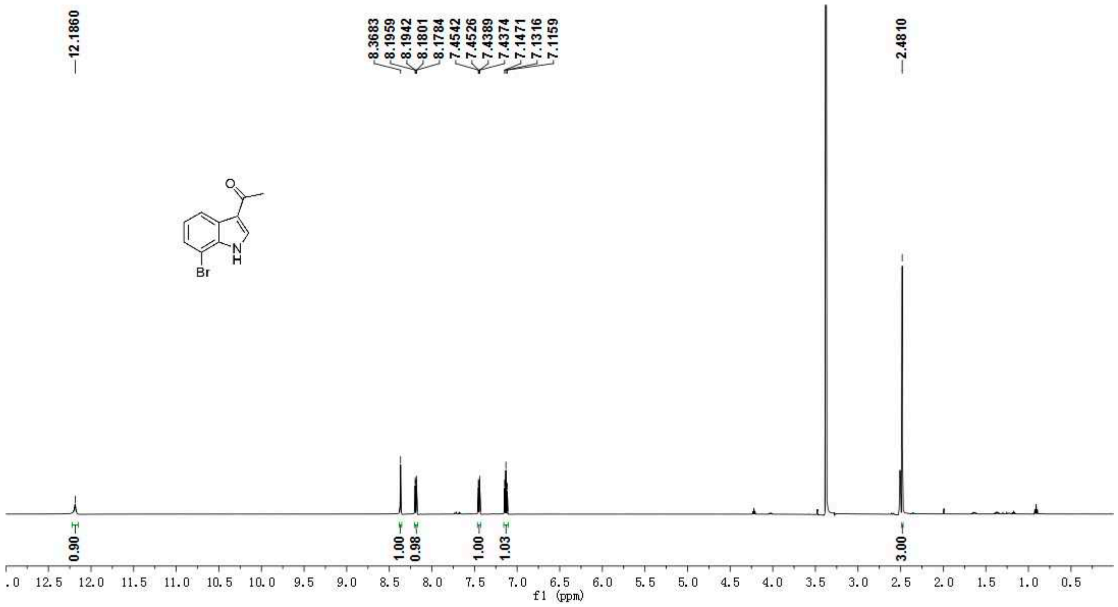

^{13}C NMR spectra (125 MHz, DMSO-d_6) of 3ja

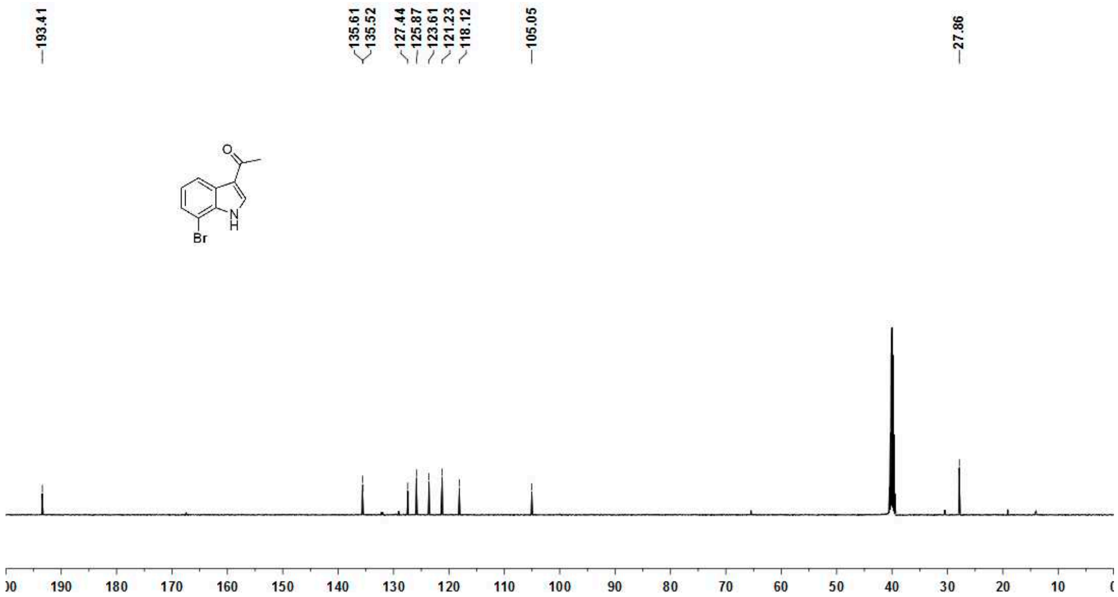

^{1}H NMR spectra (500 MHz, DMSO-d_6) of 3jb

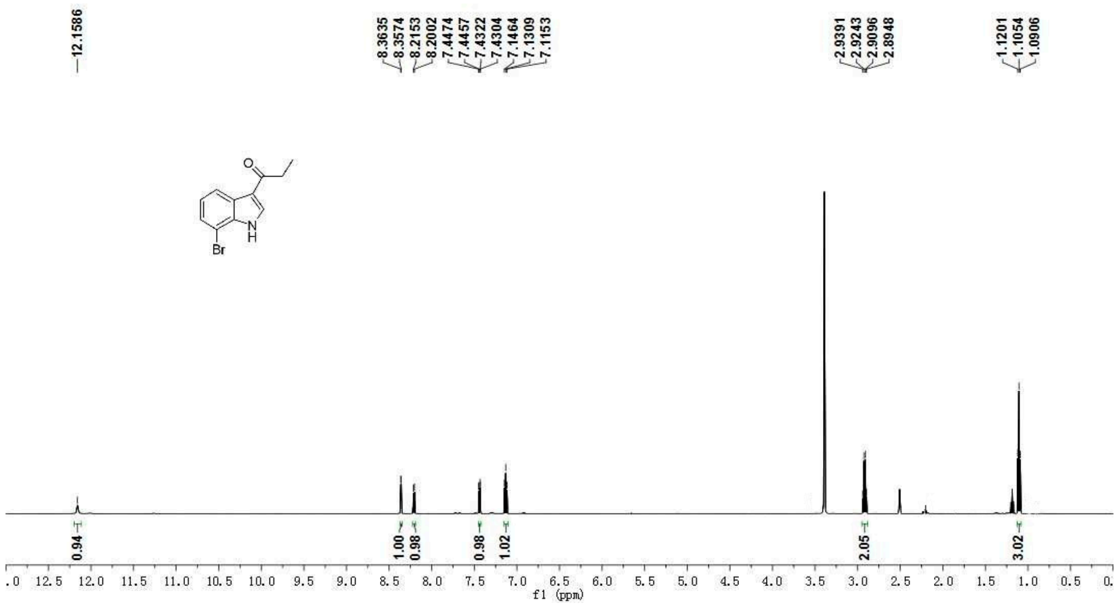

^{13}C NMR spectra (125 MHz, DMSO-d_6) of 3jb

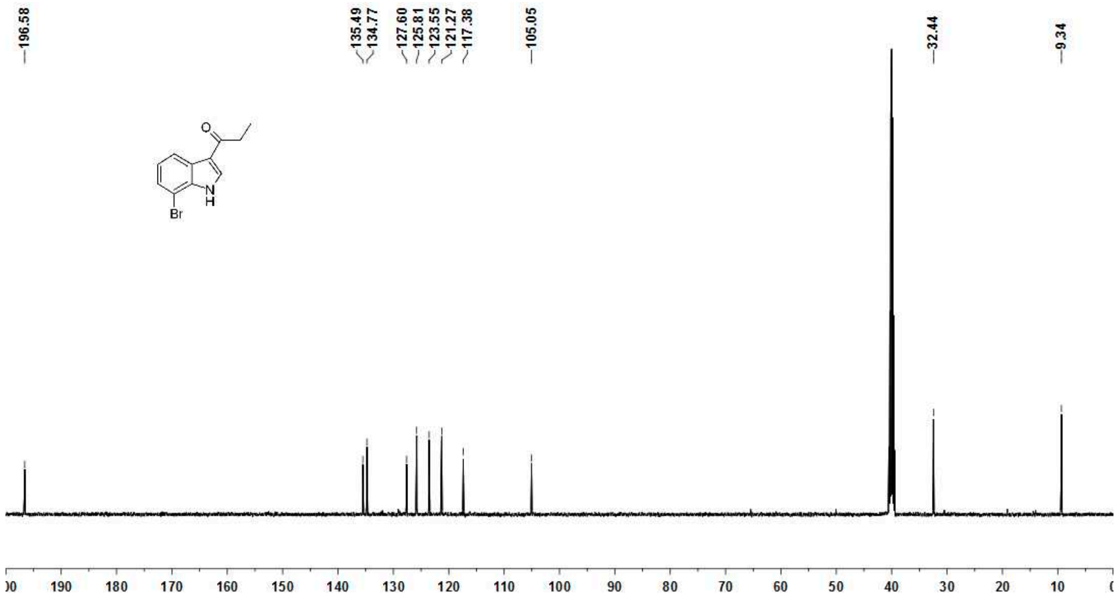

^{1}H NMR spectra (500 MHz, DMSO-d_6) of 3jc

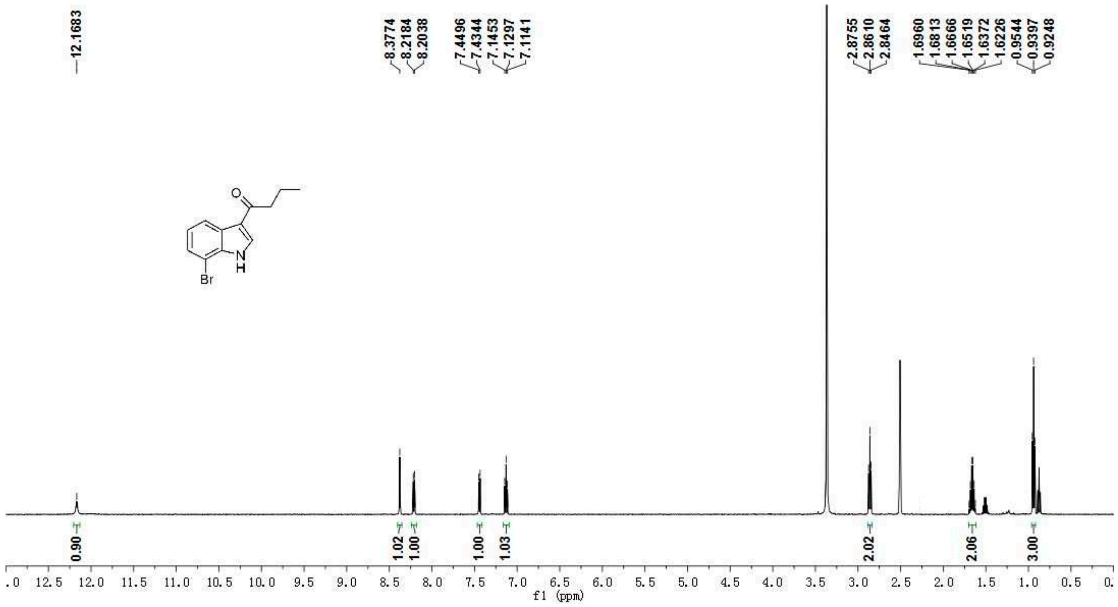

^{13}C NMR spectra (125 MHz, DMSO-d_6) of 3jc

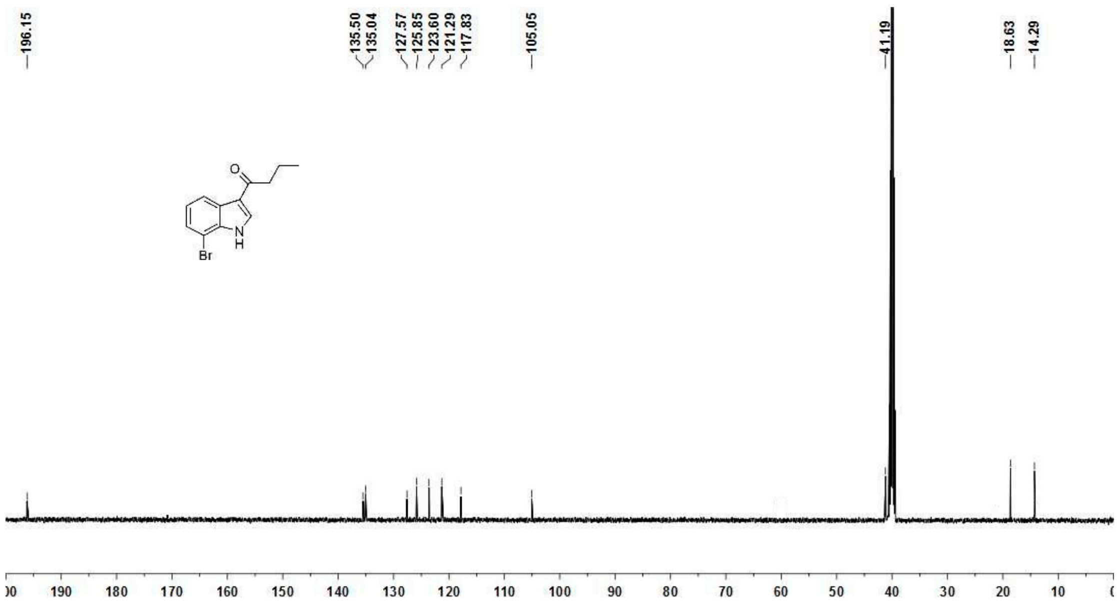

^{1}H NMR spectra (500 MHz, DMSO-d_6) of 3jd

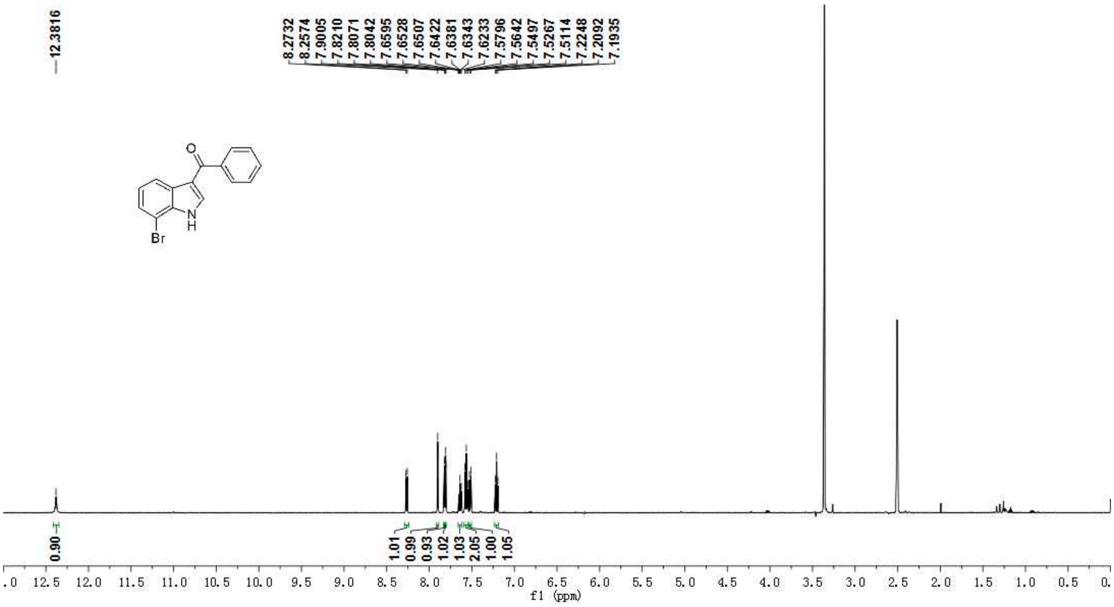

^{13}C NMR spectra (125 MHz, DMSO-d_6) of 3jd

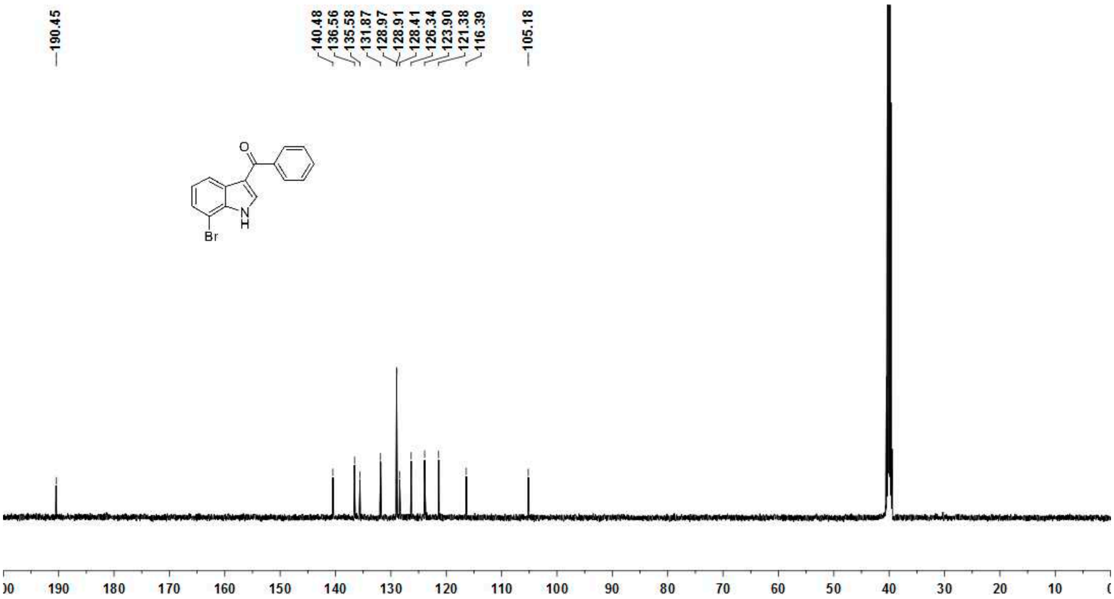

^{1}H NMR spectra (400 MHz, $CDCl_3$) of 5

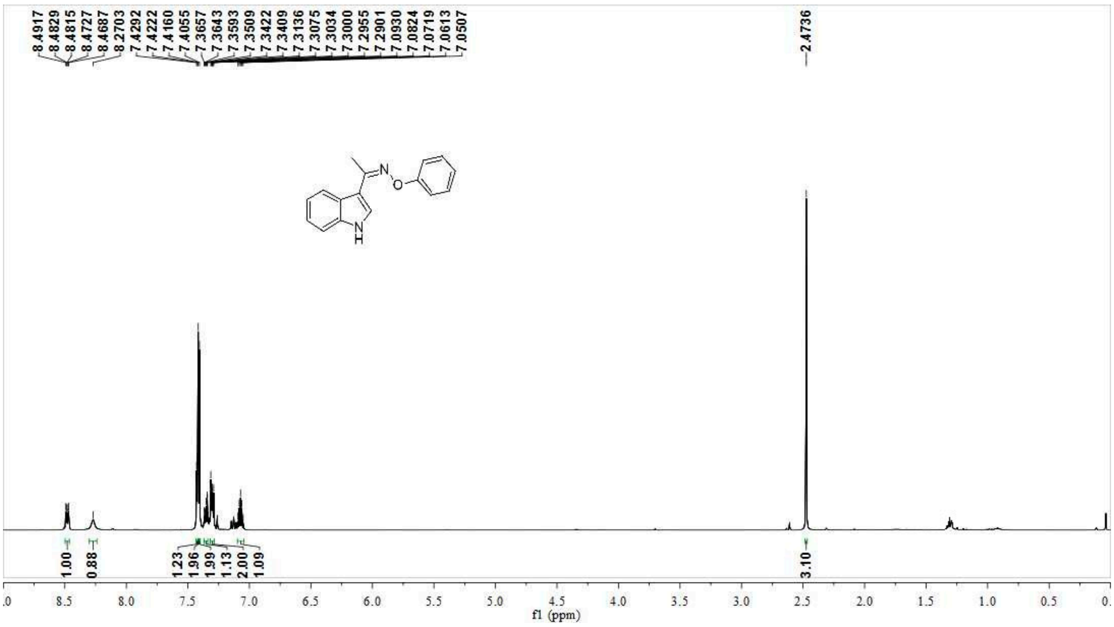

^{13}C NMR spectra (100 MHz, $CDCl_3$) of 5

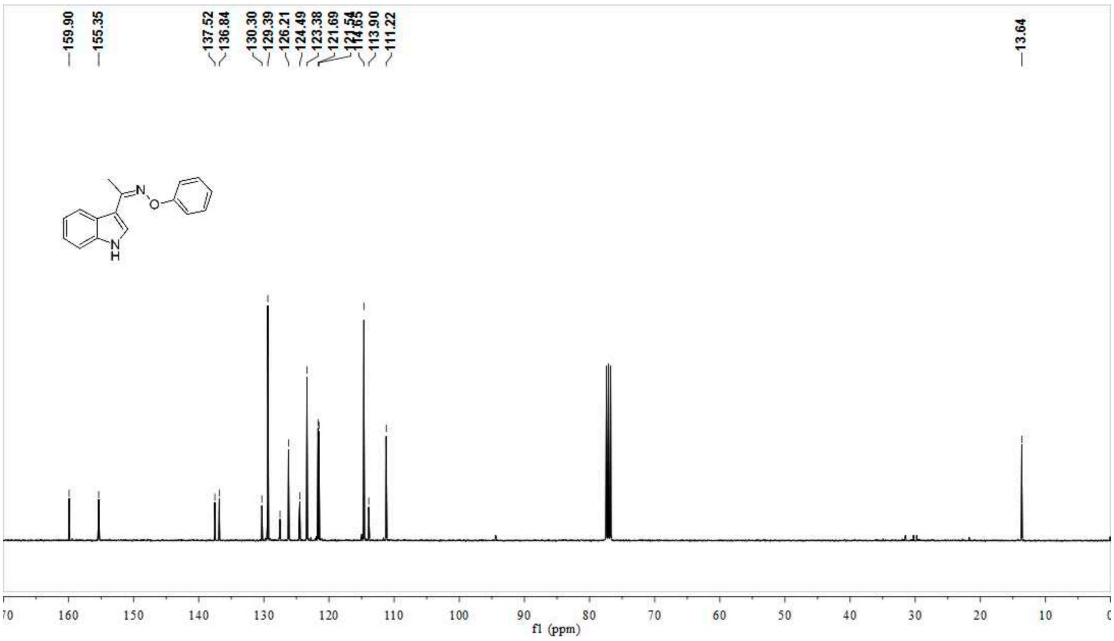

^{1}H NMR spectra (500 MHz, $CDCl_3$) of 6

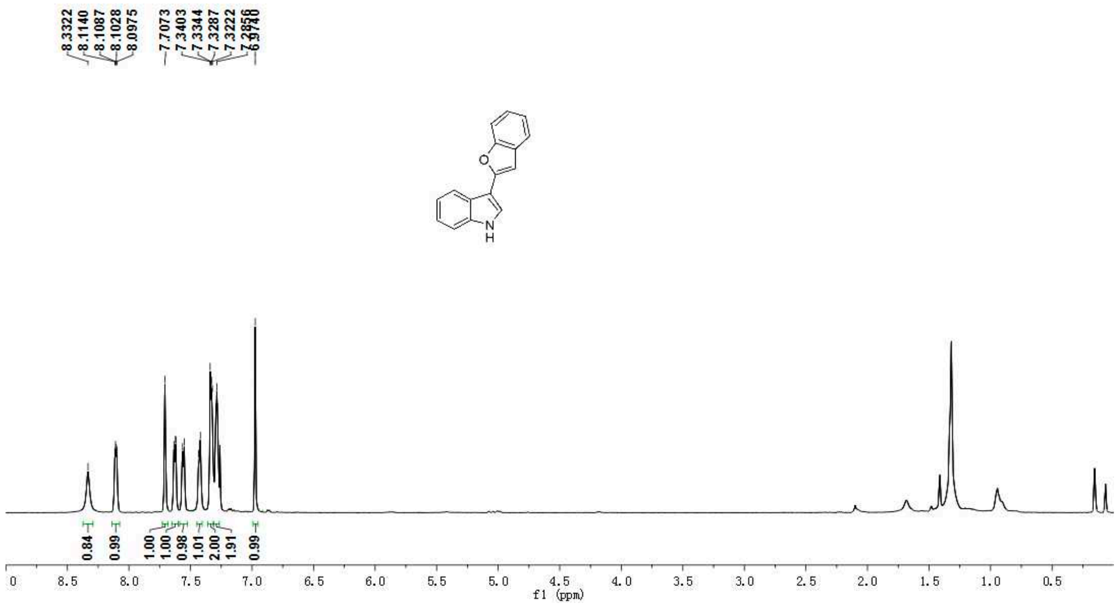

^{13}C NMR spectra (125 MHz, $CDCl_3$) of 6

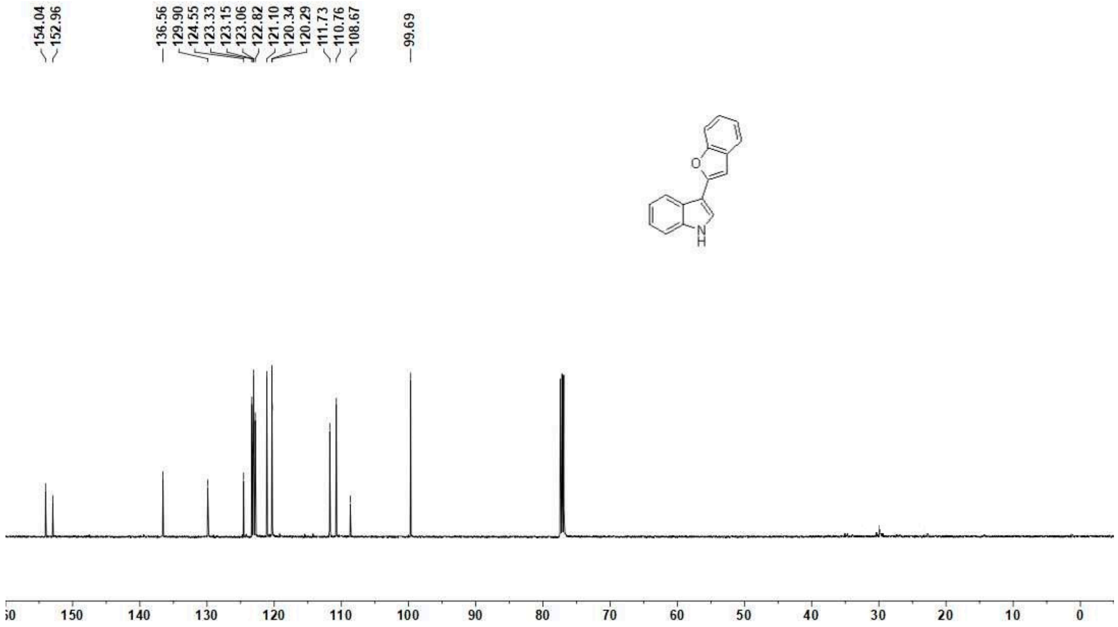

Single-Crystal X-Ray Structure of 3ec (CCDC: 1441923)

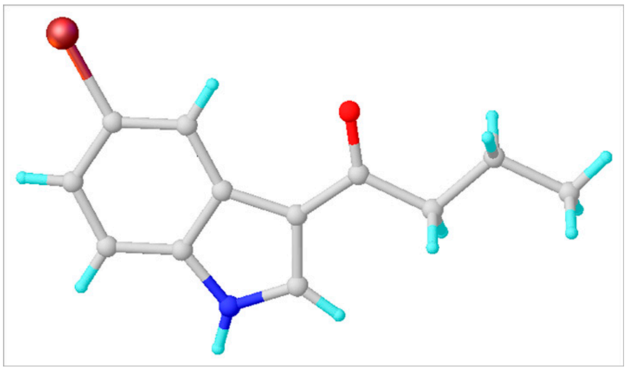

Table S1 Crystal data and structure refinement for **3ec**.

Identification code	**3ec**
Empirical formula	$C_{12}H_{12}BrNO$
Formula weight	266.14
Temperature/K	296.15
Crystal system	triclinic
Space group	P-1
a/Å	7.274(3)
b/Å	7.359(3)
c/Å	11.247(5)
α/°	85.412(7)
β/°	87.981(7)
γ/°	69.479(7)
Volume/Å^3	562.1(4)
Z	2
ρ_{calc}g/cm^3	1.572
μ/mm^{-1}	3.629
F(000)	268.0
Crystal size/mm^3	0.21 × 0.19 × 0.18
Radiation	MoKα (λ = 0.71073)
2Θ range for data collection/°	3.64 to 53.36
Index ranges	−9 ≤ h ≤ 9, −9 ≤ k ≤ 9, −14 ≤ l ≤ 14
Reflections collected	6660
Independent reflections	2357 [R_{int} = 0.0334, R_{sigma} = 0.0404]
Data/restraints/parameters	2357/0/137
Goodness-of-fit on F^2	0.841
Final R indexes [I>=2σ (I)]	R_1 = 0.0371, wR_2 = 0.1121
Final R indexes [all data]	R_1 = 0.0590, wR_2 = 0.1281
Largest diff. peak/hole / e Å^{-3}	0.55/−0.34

Table S2 Fractional Atomic Coordinates ($\times10^4$) and Equivalent Isotropic Displacement Parameters ($Å^2\times10^3$) for **3ec**. U_{eq} is defined as 1/3 of of the trace of the orthogonalised U_{IJ} tensor.

	x	*y*	*z*	U(eq)
Br1	2740.8(6)	2814.7(6)	5075.3(3)	72.1(2)
O1	2538(4)	37(3)	9744(2)	48.1(6)
N1	2633(4)	6180(3)	9755(2)	41.8(6)
C6	2628(4)	3761(4)	8657(3)	35.4(6)
C7	2502(4)	3187(4)	9902(3)	33.8(6)
C5	2623(4)	2905(4)	7594(3)	40.7(7)
C1	2731(4)	5639(4)	8607(3)	38.1(7)
C11	1949(5)	−869(5)	12151(3)	45.6(7)
C10	2374(5)	959(4)	11745(3)	42.0(7)
C8	2509(4)	4722(4)	10522(3)	39.9(7)
C12	1679(7)	−1101(6)	13496(3)	63.7(10)
C3	2928(5)	5770(5)	6494(3)	52.3(8)
C4	2761(5)	3927(5)	6542(3)	47.4(7)
C2	2916(5)	6635(4)	7535(3)	47.8(8)
C9	2475(4)	1308(4)	10411(3)	36.0(6)

Table S3 Anisotropic Displacement Parameters ($Å^2\times10^3$) for **3ec**. The Anisotropic displacement factor exponent takes the form: $-2\pi^2[h^2a^{*2}U_{11}+2hka^*b^*U_{12}+\ldots]$.

	U_{11}	U_{33}	U_{23}	U_{13}	U_{12}
Br1	93.0(4)	47.6(3)	−17.3(2)	6.4(2)	−42.9(3)
O1	78.0(15)	46.3(13)	−3.5(9)	2.6(11)	−26.2(10)
N1	56.4(15)	52.2(16)	−3.7(10)	−2.3(12)	−20.8(11)
C6	37.0(14)	48.0(17)	−1.1(11)	0.0(12)	−12.7(11)
C7	40.7(15)	41.8(16)	−1.1(11)	−0.3(12)	−13.7(11)
C5	46.9(16)	47.3(18)	−5.3(12)	1.5(13)	−17.5(13)
C1	41.4(16)	50.1(18)	−2.0(12)	−3.0(13)	−15.0(12)
C11	59.5(19)	43.1(18)	5.6(13)	−4.3(14)	−24.7(15)
C10	53.1(18)	44.7(17)	−2.7(12)	0.5(13)	−18.7(13)
C8	48.4(17)	45.1(17)	−2.6(12)	0.9(13)	−19.5(13)
C12	91(3)	45(2)	9.3(17)	−3.7(18)	−41(2)
C3	63(2)	49(2)	14.0(15)	−0.4(15)	−26.2(16)
C4	51.4(18)	43.9(18)	−4.9(14)	3.0(14)	−20.5(15)
C2	62(2)	54(2)	7.2(14)	−2.9(16)	−26.1(15)
C9	41.5(15)	45.4(17)	−0.2(12)	0.0(12)	−15.0(11)

Table S4 Bond Lengths for **3ec.**

		Length/Å			Length/Å
Br1	C4	1.901(4)	C7	C9	1.460(4)
O1	C9	1.233(4)	C5	C4	1.371(4)
N1	C1	1.373(4)	C1	C2	1.388(4)
N1	C8	1.348(4)	C11	C10	1.516(4)
C6	C7	1.440(4)	C11	C12	1.522(5)
C6	C5	1.395(4)	C10	C9	1.505(4)
C6	C1	1.407(4)	C3	C4	1.401(5)
C7	C8	1.375(4)	C3	C2	1.375(5)

Table S5 Bond Angles for **3ec.**

			Angle/°				Angle/°
C8	N1	C1	109.5(2)	C10	C11	C12	113.0(3)
C5	C6	C7	134.7(3)	C9	C10	C11	114.0(3)
C5	C6	C1	119.1(3)	N1	C8	C7	110.0(3)
C1	C6	C7	106.1(3)	C2	C3	C4	119.6(3)
C6	C7	C9	126.7(2)	C5	C4	Br1	119.3(2)
C8	C7	C6	106.5(2)	C5	C4	C3	122.8(3)
C8	C7	C9	126.7(3)	C3	C4	Br1	117.9(3)
C4	C5	C6	118.1(3)	C3	C2	C1	118.3(3)
N1	C1	C6	107.9(3)	O1	C9	C7	119.6(3)
N1	C1	C2	130.1(3)	O1	C9	C10	121.2(3)
C2	C1	C6	122.0(3)	C7	C9	C10	119.2(2)

Table S6 Hydrogen Atom Coordinates (Å×10[4]) and Isotropic Displacement Parameters (Å[2]×10[3]) for **3ec.**

	x	*y*	*z*	U(eq)
H1	2648	7279	9953	50
H5	2528	1676	7600	49
H11A	3023	−1992	11901	55
H11B	768	−839	11762	55
H10A	1358	2068	12058	50
H10B	3613	874	12082	50
H8	2438	4751	11347	48
H12A	1452	−2296	13704	96
H12B	575	−29	13745	96
H12C	2840	−1125	13887	96
H3	3046	6406	5762	63
H2	3029	7857	7521	57
